# Geniposide Alleviates Inflammatory Bowel Disease by Regulating Intestinal Flora and Arginine Metabolism and Inhibiting the NF‐κB Pathway Through Targeting Anxa5

**DOI:** 10.1155/mi/6231832

**Published:** 2026-07-23

**Authors:** Xiu Wang, Francis Atim Akanyibah, Pengfei Zhang, Xiaodong Zhou, Ming Zhang, Fei Mao

**Affiliations:** ^1^ Department of Laboratory Medicine, The Affiliated People’s Hospital, Jiangsu University, Zhenjiang 212013, Jiangsu, China, ujs.edu.cn; ^2^ Institute of Hematology, Jiangsu University, Zhenjiang 212013, Jiangsu, China, ujs.edu.cn; ^3^ Department of Anatomy, School of Medicine and Dentistry, Entrance University of Health Sciences, 16 Okpoi Gonno Spintex, Accra, Ghana; ^4^ Department of General Surgery, The Affiliated People’s Hospital, Jiangsu University, Zhenjiang 212002, Jiangsu, China, ujs.edu.cn; ^5^ Department of Laboratory Medicine, Changzhou Maternal and Child Health Care Hospital, Changzhou 213300, Jiangsu, China

**Keywords:** Anxa5, arginine metabolism, geniposide, gut microbiota, inflammatory bowel disease, NF-κB

## Abstract

**Background:**

Inflammatory bowel disease (IBD), including Crohn’s and ulcerative colitis (UC), is a chronic gut inflammation thought to be caused by gut microbial causes and immune system dysfunction. Geniposide (GEN) is a natural compound shown to prevent IBD; however, its mechanism of modifying the gut microbiome, regulating arginine metabolism, and inhibiting the nuclear factor kappa B (NF‐κB) pathway by targeting Annexin A5 (Anxa5) remains unexplored.

**Methods:**

This study examined the regulation of intestinal immunity, gut microbiota, metabolites, and associated activities and pathways, as well as the NF‐κB pathway by GEN in a BALB/c mouse model of IBD. The mouse macrophage cell line (RAW264.7) was further utilized to investigate the role of GEN on the macrophage‐arginine metabolism and the Anxa5 axis. qRT‐PCR, Western blotting, hematoxylin and eosin (H&E), immunohistochemistry (IHC), immunofluorescence (IF), fecal 16S rDNA sequencing, and UHPLC/Q‐TOF‐MS were used to evaluate the treatment effect of GEN. Drug target sequencing and small interfering RNA (siRNA) were used to establish the target molecule of GEN.

**Results:**

GEN therapy improved colon and spleen tissues, decreased the disease activity index, helped mice maintain their weight, elevated anti‐inflammatory cytokines and tight junction proteins, and decreased proinflammatory cytokines. GEN modulated the quantity of dysfunctional metabolites and enhanced the structure and diversity of the gut microbial community. GEN restored the underpopulated genera *Enterorhabdus*, *Rikenella*, *Anaerotruncus*, and *Alistipes*, regulating arginine metabolism mediated by G‐guanidinobutyrate in RAW264.7 cells and the gut mucosa to protect the colon. GEN targeted Anxa5, enhancing its expression in both animal models and RAW264.7 cells. Following Anxa5 knockdown, cyclooxygenase‐2 (COX‐2) expression increased, along with the activation of NF‐κB pathway–related proteins in RAW264.7 cells. GEN reduced the levels of NF‐κB pathway–related proteins in both RAW264.7 cells and animal models.

**Conclusion:**

GEN mitigated colitis by regulating the intestinal microbiota and arginine metabolism and inhibiting the NF‐κB pathway via targeting Anxa5.

## 1. Introduction

Inflammatory bowel diseases (IBDs), including ulcerative colitis (UC) and Crohn’s disease (CD), are defined by chronic intestinal inflammation, which is linked to increased intestinal epithelial cell (IEC) death [1, 2]. This erosion of the gut barrier triggers immunological responses and leads to further IEC death [1]. IBD has become prominent in Western countries, and its frequency is rapidly increasing in newly industrialized countries [3]. Abdominal pain, diarrhea, bleeding, fever, and weight loss are some of the symptoms that may occur, depending on the location, type, and severity of the condition [4]. An uncontrolled immune response to dysbiosis of the gut microbiota is a contributing factor to IBD [5]. Reduced biodiversity, abnormal gut microbiota composition, altered geographic distribution, and interactions among microbiota, between microbiota strains, and with the host are all indicators of microbial dysbiosis [6]. Furthermore, metabolites are among the primary mechanisms by which the gut microbiota communicate with the host [7]. As a result, microbial dysbiosis can lead to unregulated metabolites, and agents that can improve dysbiosis and gut microbiota metabolites may be useful for IBD treatment.

Macrophages play an important function in IBD and are the main defenders of intestinal immunological homeostasis [8]. Research indicates that M1 macrophages contribute to the development of IBD, whereas M2 macrophages help to alleviate its symptoms. Consequently, substances that promote the polarization of M2 macrophages may be beneficial for recovery from IBD [9]. Additionally, M2 macrophages interact with the microbiota, repair intestinal tissue, and decrease inflammation [10, 11]. The anti‐inflammatory effects of macrophages have been linked to arginine metabolism–related molecules, where arginase 1 (Arg1) and inducible nitric oxide synthase (iNOS) define alternatively activated M2 macrophages and traditionally activated M1, respectively [12]. Arginine metabolism is crucial in the pathophysiology of IBD, as its constituents, the arginine‐polyamine and arginine‐creatine axes, primarily inhibit inflammation, unlike the proinflammatory iNOS axis [13]. Additionally, creatine rewires macrophage polarization by boosting IL‐4‐STAT6–activated Arg1 expression and blocking immunological effector molecules, including iNOS and IFN‐γ‐JAK‐STAT1 transcription factor signaling. Reduced expression of solute carrier family (SLC) 6A8 depletes intracellular creatine, which affects immunological responses mediated by macrophages [14]. Also, SLC 7 member 2 (SLC7A2) is an arginine transporter, and the high‐affinity transporter SLC7A2B is widely found in macrophages [15, 16]. SLC7A2 and Arg1 mRNA levels are lower in active IBD, but Arg2 and NOS2 mRNA levels are higher [17]. As a result, medications that can regulate arginine metabolism by boosting Arg1, SLC7A2, and SLC 6 member 8 (SLC6A8) and inhibiting iNOS may help prevent IBD.

Additionally, nuclear factors, particularly nuclear factor kappa B (NF‐κB), can be activated in IBD, leading to increased transcription of proinflammatory mediators. This activation results in diarrhea, stomach pain, bleeding, and various extraintestinal symptoms [18]. NF‐κB pathways are among the numerous inflammatory targets that phytochemicals can disrupt [18]. The phosphorylation of NF‐κB p65 in prostate cancer cells increases when Annexin A5 (Anxa5) is knocked down, indicating that Anxa5 suppresses p65 activation, which results in cyclooxygenase‐2 (COX‐2) inhibition [19], suggesting that targeting Anxa5 could block the NF‐κB pathway. Moreover, studies have shown that Anxa5 may influence macrophage polarization from the M1 to the M2 phenotype in the liver [20] and in diabetic wound healing and inflammation control [21]. Jia et al. [22] also found that by promoting the internalization and lysosomal degradation of toll‐like receptor (TLR) 4 via calcium‐dependent endocytosis, Anxa5 effectively prevented M1 macrophage polarization and reduced the release of proinflammatory mediators. In arginine metabolism, M1 macrophages express the enzyme NOS, which transforms arginine into nitric oxide (NO) and citrulline. M2 macrophages, conversely, express arginase, which catalyzes the conversion of arginine into ornithine and urea [23]. Thus, agents targeting Anxa5 can polarize the M2 phenotype and switch arginine metabolism to the arginase pathway to prevent inflammation.

Geniposide (GEN), a compound primarily extracted from *Gardenia jasminoides* Ellis and contained in traditional phytomedicines, has proven crucial for new drug development in recent years [24, 25]. Studies have shown that GEN can ameliorate IBD via the nuclear factor–like erythroid 2–related factor 2/ARE signaling [26], AMPK signaling pathway [27], and poly ADP–ribose polymerase 1/PI3K/AKT signaling pathway–suppressed lipophagy [28]. The role of GEN in modulating gut microbiota, macrophage arginine metabolism, and the NF‐κB pathway to alleviate IBD by targeting Anxa5 remains unknown. This study investigated how GEN affected mucosal tissue repair, gut microbiota structure and metabolites, arginine metabolism, NF‐κB pathway proteins, and Anxa5 in macrophages and an IBD animal model.

## 2. Materials and Methods

### 2.1. GEN

GEN, with the molecular formula C_17_H_24_0_10_, was acquired from Sichuan Vikki Biotechnology Co., Ltd., with CAS Number: 24512‐63‐8.

### 2.2. Establishment of the Animal Model

Male BABL/c mice weighing 20 ± 2 g and aged 6–8 weeks were acquired from Jiangsu University’s Animal Research Center in Zhenjiang, China. The animal model investigation was conducted in compliance with housekeeping and animal care standards. The mice were acclimatized (2 days) before the main experiments. Mice were healthy with no signs of illness. A total of 15 mice were assigned at random to three categories: a negative control (*n* = 5), an IBD model triggered by dextran sulfate sodium (DSS) (*n* = 5), and a therapy group (DSS‐induced injury + GEN) (*n* = 5). The sample size was determined based on a previous experiment published by Xu et al. [29]. The mice were housed in a 12‐h light/dark cycle (12 h of light during the day and 12 h of darkness at night) and provided with adequate ventilation. The mice in the negative control group consumed sterile water under high pressure; those in the DSS‐induced IBD model and treatment group (DSS‐induced injury + GEN) drank a mixture of 3% DSS water and sterile water under high pressure. The DSS‐induced injury + GEN group received a GEN solution (200 mg/kg) by gavage daily, while the DSS‐induced IBD models received the same volume of sterile water via gavage at the same time each day. A dose of 200 mg/kg was chosen for treatment based on a previous study by Zhang et al. [30], and a pilot study was conducted to confirm its effectiveness in mitigating colitis. The DSS/GEN treatment lasted for 10 days. DSS‐induced IBD evaluation included daily weighing of the mice, monitoring fecal changes, measuring daily feed consumption, assessing daily water intake, and conducting pathological analysis using a disease activity index (Table 1). When the DSS group’s mice showed bloody stool and weight loss, fecal samples from all groups were collected under aseptic conditions for UHPLC/Q‐TOF–MS analysis and 16S rDNA sequencing. On day 10, every mouse was euthanized using gas anesthesia (isoflurane). A total of 20 mL of isoflurane was introduced into the gas anesthesia machine. The mice were then placed in a sealed box connected to the machine, leading to their death under gas anesthesia. This procedure follows the ARRIVE guidelines 2.0. Following anesthesia, the splenic, intestinal, and rectal tissues were separated for further testing and analysis.

**Table 1:** The disease activity index (DAI) score.

Values	Weight loss (%)	Fecal consistency	Bowel bleeding
0	<1	Normal hard stool	No bleeding
1	1–5	Soft stool	Blood in stool
2	5–10		
3	10–15	Moderately soft stool	Dominant bleeding
4	>15	Loose stool, completely uniformed	

*Note:* DAI = (weight loss (%) + stool consistency + rectal bleeding)/3.

### 2.3. Cell Culture

The mouse leukemic monocyte/macrophage cell line (RAW264.7) was obtained from Procell (Wuhan, China) and cultivated in DMEM (Hyclone, US) comprising 10% fetal calf serum (FBS; Excell, Uruguay) at 37°C in humid air with 5% CO_2_. RAW264.7 cells (1 × 10^6^) were seeded in a 12‐well plate/well, and when the cells reached a density of about 60%–80%, an inflammatory milieu caused by lipopolysaccharide (LPS) (1 µg/mL) was created, and treatment including or excluding GEN (50 µM) was administered for 24 h. A concentration of 1 µg/mL LPS was selected based on studies by Xia et al. [31] and Zhou et al. [32], which successfully created an inflammatory condition in RAW264.7 cells. Additionally, a treatment dose of 50 µM of GEN was selected because it was not toxic to the cells and it effectively reduced TNF‐α levels in RAW264.7 cells treated with LPS, compared to other doses (5, 10, and 30 µM of GEN).

### 2.4. CCK8

RAW264.7 cells were separated into 96‐well plates and administered with varied doses of GEN (5, 10, 30, and 50 µM) to evaluate GEN toxicity on the cells. CCK8 solution (Vazyme, China) was added to the culture wells. A microplate reader from Thermo Fisher Scientific (China) was used to measure the absorbance at 450 nm after an hour of incubation in the dark at 37°C.

### 2.5. Micromethod Detection of L‐Arginine (Arg), Creatine, and NO

#### 2.5.1. Arg and Creatine

RAW264.7 cells were seeded in a 6‐well plate/well after being grown in full media (DMEM with 10% FBS), followed by incubation at 37°C in humid air with 5% CO_2_. Once the cells reached 60%–80% density, LPS at 1 µg/mL was administered, along with treatments that included or excluded GEN (50 µM), for a 24‐h incubation. The cells were then collected and placed in EP tubes using cell lysis solution. One milliliter of extract solution I (Beijing Solarbio Science & Technology Co., Ltd., China) was administered to the treated RAW264.7 cells. The cells were disrupted in an ultrasonic ice bath (300 W, 3 s ultrasonic signal, 7 s interval, for a total duration of 3 min) before centrifugation for 10 min at 12,000 g and 4°C. 0.8 mL of clear liquid was taken, and 0.15 mL of extract solution II (Beijing Solarbio Science & Technology Co., Ltd., China) was added slowly, mixed, and blown until no bubbles were produced. Following 10 min of centrifugation at 12,000 g and 4°C, an enrichment sample was taken for microplate readings (Thermo Fisher Scientific, China) at 525 nm (Arg) and 530 nm (creatine).

#### 2.5.2. NO

The supernatant from treated RAW264.7 cells was collected for NO measurement using an NO testing kit (Beyotime, China). In accordance with the kit protocol, both the sample and standard preparations were added to a 96‐well plate at 50 µL/well. Fifty microliters of room‐temperature Griess reagent (I) was added to each well. Additionally, 50 µL of Griess reagent (II) at room temperature was applied to every well, and the absorbance at 540 nm was determined utilizing the multipurpose microplate detector (Thermo Fisher Scientific, China). Nitrite was used as the standard for NO. A graph was plotted with the concentration of sodium nitrite (NaNO_2_) on the *X*‐axis and the absorbance on the *Y*‐axis to determine a standard curve. Then, the concentration of NO in the samples in various groups (NC, LPS, and GEN) was determined using the absorbance determined at 540 nm based on the standard curve.

### 2.6. Flow Measurement of Cell Cycle

To determine if GEN can maintain a normal cell cycle under inflammatory conditions, treated cultured cells were centrifuged, and the supernatant was collected and washed with PBS. The resuspended cells were treated with absolute ethanol at −20°C and then stored overnight at this temperature. The cells underwent centrifugation, washing, centrifugation, and supernatant collection. The supernatant was treated with 100 µL of RNase reagent and suspended in a water bath at 37°C for 30 min. The cells were then treated with 400 µL PI and incubated for 30 min. Fluorescence detection at 488 nm was done using flow cytometry.

### 2.7. Quantitative Real‐Time Polymerase Chain Reaction (qPCR)

qPCR was used to evaluate the total RNA obtained from colon samples and RAW264.7 cells after purification with RNA total cleanup kits according to the manufacturer’s instructions. The HiScript II 1st Strand cDNA Synthesis Kit (Vazyme Biotech Co., Ltd.) was used first, followed by the AceQ Universal SYBR Green qPCR Master Mix. Target gene expression was measured using β‐actin as an internal reference. Annealing temperatures and the number of amplification cycles were set to the required values to provide an acceptable read, as specified by the manufacturer. Table 2 shows the primer sequences used.

**Table 2:** Sequence of primers used for qRT‐PCR.

Primer name	Primer sequence (5′→3′)	Annealing temperatures (°C)
Mouse‐β‐actin	F: GCTCCGGCATGTGCAAAG	60
	R: TTCCCACCATCACACCCTGG	
Mouse‐TNF‐α	F: CCCTCACACTCACAAACCAC	60
	R: ACAAGGTACAACCCATCGGC	
Mouse‐IL‐1β	F: ATGCCACCTTTTGACAGTGATG	60
	R: TGATGTGCTGCTGCGAGATT	
Mouse‐IL‐6	F: TTTCCTCTGGTCTTCTGGAGT	60
	R: TCTGTGACTCCAGCTTATCTCTTG	
Mouse‐IL‐10	F: TGAATTCCCTGGGTGAGAAGC	60
	R: CACCTTGGTCTTGGAGCTTATT	
Mouse‐iNOS	F: ATCTTGGAGCGAGTTGTGGATTGTC	60
	R: TAGGTGAGGGCTTGGCTGAGTG	
Mouse‐SLC7A2	F: TTGCCGTGTGCCTTGTATTACTCC	60
	R: AAACCCAGCCACCATGACAAAGAG	
Mouse‐SLC6A8	F: CTTGGACACGCCAGATGGACTTC	60
	R: CGCCGTTCTTGTAGCACAGGTAG	
Mouse‐Arg1	F: AATCTGGTTGTGTATCCTCGTT	60
	R: AGAGGTGTATTAATGTCCGCAT	
Mouse Anax5	F: CATCGTAGAGTCGTGAGGGC	60
	R: GGGACCTTGTGGATGACCTG	
Mouse COX‐2	F: AACACCTGAGCGGTTACCACTTC	60
	R: AGAGGCAATGCGGTTCTGATACTG	

### 2.8. Western Blotting

Colon mucosa and RAW264.7 cells were lysed in RIPA lysis buffer and supplemented with proteinase inhibitors (Solarbio, Beijing, China). Protein samples were then subjected to gradient separation by 10% sodium dodecyl sulfate–polyacrylamide gel electrophoresis, and then the separated proteins were transferred to polyvinylidene fluoride membranes (Merck Millipore, Tullagreen, Carrigtwohill, Co. Cork, Ireland). Membranes were blocked with 5% milk in Tris‐buffered saline‐Tween 20 and incubated with the corresponding primary antibodies of target proteins according to the following ratio: iNOS (1:500) (Proteintech, catalog number: 22226‐1‐AP, China), SLC7A2 (1:800) (Proteintech, catalog number: 30232‐1‐AP, China), SLC6A8 (1:500) (Proteintech, catalog number: 20299‐1‐AP, China), Arg1 (1:5000) (Proteintech, catalog number: 16001‐1‐AP, China), β‐actin (1:1000) (ABclonal, catalog number: AC006, China), COX2 (1:1000) (Abcam, catalog number: ab179800, UK), p‐IκBα (1:1000) (CST, catalog number: 2859T, US), IκBα (1:1000) (CST, catalog number: 4814T, US), p‐P65 (1:1000) (CST, catalog number: 3033 S, US), P65 (1:1000) (CST, catalog number: 8242S, US), Annexin V (1:1000) (Santa Cruz, catalog number: sc‐74438, China), occludin (1:5000) (Proteintech, catalog number: 27260‐1‐AP, China), PCNA (1:800) (Abcam, catalog number: ab29, UK), and claudin‐1 (1:1000) (Proteintech, catalog number: 28674‐1‐AP, China). After being mixed with the primary antibody for at least 6 h at 4°C, the blots were treated with separate secondary antibodies, goat antimouse IgG (H + L) (HRP) (Immunoway, catalog number: RS0001, USA) and goat antirabbit IgG (H + L) (HRP) (Immunoway, catalog number: RS0002, USA), for 1 h at 37°C and then made visible using chemiluminescence (A and B) (Abbkine, USA), prepared in a 1:1 ratio, and detected with gel imaging software (GE Healthcare Life Sciences, USA). ImageJ was used to quantify the Western blot band intensities for statistical analysis.

### 2.9. Hematoxylin and Eosin (H&E) Staining

To analyze structural damage to the colonic mucosa and spleen and the reparative effect of GEN at the tissue level, H&E staining was conducted using standardized protocols. PBS was used to wash the fresh colorectal and spleen tissues in mice one to two times. The tissues were fixed in 4% paraformaldehyde, embedded in paraffin, and subsequently sectioned utilizing a microtome. The sections were mounted on a slide, dewaxed, stained with H&E, and then scanned and examined using a pathological biopsy scanner.

### 2.10. Immunohistochemistry (IHC)

Tissue sections were dried for at least 6 h in an oven set to 56°C. They were then dewaxed using xylene and a gradient of ethanol concentrations. To block the endogenous peroxidase activity, a 3% hydrogen peroxide solution was applied. Subsequently, the hot‐fixed antigen retrieval was done using freshly prepared 0.01 M citrate buffer. Finally, they were blocked for 30 min at room temperature using 5% BSA. Following 5% BSA blocking, they were incubated overnight at 4°C with primary antibodies SLC7A2 (Proteintech, catalog number: 30232‐1‐AP, China), SLC6A8 (Proteintech, catalog number: 20299‐1‐AP, China), and Arg1 (Proteintech, catalog number: 16001‐1‐AP, China) at dilution ratios of 1:200, 1:200, and 1:800, respectively. The secondary antibody, HRP polymer (Vazyme, catalog number: HC301, China), was then applied for 30 min at room temperature. The sections were incubated with a diaminobenzidine (DAB) substrate (DAB preparation, 100 μL DAB solution (10×) to 1 mL of DAB buffer (Vazyme, catalog number: HC301, China)), counterstained with hematoxylin, dehydrated, and sealed with a neutral resin for microscopic analysis.

### 2.11. Immunofluorescence (IF)

#### 2.11.1. Tissue

For tissue IF, tissue fixation, embedding, thermal antigen retrieval, and application of BSA solution were performed similarly to the IHC procedure. Following 5% BSA blocking, the sections were allowed to incubate with an iNOS primary antibody (1:100) (Proteintech, catalog number: 22226‐1‐AP, China) for the entire night at 4°C. The sections were incubated with the fluorescent secondary antibody FITC (BOSTER, catalog number: BA1105, China) at a 1:100 dilution for 2 h at room temperature. 4’,6‐Diamidine‐2‐phenylindole (DAPI), a quenching agent, was added drop by drop onto each section after washing, and the sectioned tissues were covered with clean coverslips to prevent air bubbles and fixed with nail polish. The sections were examined using a Nikon fluorescence microscope (Japan).

#### 2.11.2. Cell

RAW264.7 cells from the culture were rinsed with PBS and fixed in 4% paraformaldehyde for 30 min at room temperature. 0.1% Triton X‐100 was added and mixed gently for 30 min, followed by blocking nonspecific antigens with a 5% BSA solution for half an hour. The cells were treated with the primary antibodies iNOS (1:250) (Proteintech, catalog number: 22226‐1‐AP, China), SLC7A2 (1:250) (Proteintech, catalog number: 30232‐1‐AP, China), SLC6A8 (1:250) (Proteintech, catalog number: 20299‐1‐AP, China), and Arg1 (1:250) (Proteintech, catalog number: 16001‐1‐AP, China) for an entire night at 4°C. The samples were subsequently incubated with fluorescent secondary antibodies FITC (1:100) (BOSTER, catalog number: BA1105, China) and CL594 (1:200) (Proteintech, catalog number: SA00013‐3, China) at 37°C for 60 min. DAPI staining was applied to the nuclei, and the findings were captured and analyzed using a Nikon fluorescence microscope (Japan).

### 2.12. 16S rDNA Gene Sequencing

DNA was isolated from the excrement of various mouse groups and then amplified using PCR, and the products were purified. As a result, a library was established. The variable segment V3–V4 of the DNA was amplified and subjected to sequencing on the Illumina NovaSeq 6000 platform. Species classification analysis was conducted on the resulting data.

#### 2.12.1. Microbial Species Analysis

The statistical method in the microbial datasets included the community structure analysis determining the community abundance of each group; diversity analysis (Wilcoxon rank‐sum test) including α diversity (based on the Chao1 index) and β diversity (based on unweighted UniFrac beta distances) to analyze the diversity of microbial communities within the sample and compare the microbial community composition of different samples, respectively; principal component analysis (PCA) to determine the similarity of the community compositions; species difference analysis (STAMP and LEfSe), where LEfSe was used for the quantitative analysis of biomarkers within different groups and the STAMP difference analysis (STAMP *t*‐test) was used to compare the species abundance between two groups of samples; and function prediction analysis (KEGG functional prediction and differential analysis) was used to determine the bacterial function differences between the groups.

### 2.13. Untargeted Metabolomics Detection

A mixture of methanol, acetonitrile, and water that has been precooled (2:2:1, v/v) was applied to a suitable volume of mouse feces. Following vortexing and low‐temperature ultrasonication, the mixture was centrifuged to obtain the supernatant, which was then vacuum‐dried. For LC‐MS analysis, the dried sample was reconstituted with acetonitrile/water (1:1, v/v), and the supernatant was extracted by centrifugation. The metabolites’ composition was determined by comparing MS/MS spectra and exact m/z values (<10 ppm) against a data repository. Data analysis was then conducted to identify compounds with significant differences.

#### 2.13.1. LC‐MS/MS Analyses

In Zhongke New Life Biotechnology Co., Ltd. and Applied Protein Technology Co., Ltd., analyses were performed using a UHPLC (1290 Infinity LC, Agilent Technologies) coupled to a quadrupole time‐of‐flight mass spectrometer (AB Sciex TripleTOF 6600).

### 2.14. Drug Target Sequencing

RAW264.7 cells were divided into two groups: LPS and LPS‐GEN, each containing three samples. The processes involved included protein lysis and extraction → drug and protein incubation → limited enzymatic digestion → enzymatic digestion → DIA proteomics data processing. Following the experiment, the data were searched in a database, and bioinformatics analyses were performed, including quantitative identification, abundance difference analysis, feature sequence analysis, and related analyses.

### 2.15. Small Interfering RNA (siRNA) Validation and Treatment Procedure

RAW264.7 cells were planted into a 12‐well plate, one per sample, after being grown in full media (DMEM with 10% FBS). After 12 h of incubation, different siRNAs (siRNA‐1: Anxa5‐mus‐416, siRNA‐2: Anxa5‐mus‐647, and siRNA‐3: Anxa5‐mus‐777, GenePharma, China) were added to each well and incubated for 48 h. After 48 h, the cells were harvested, and Western blotting and qRT‐PCR were used to assess siRNA efficacy in knocking down Anxa5 (Santa Cruz, China). After validating the siRNA that effectively knocked down Anxa5 (siRNA‐3: Anxa5‐mus‐777, GenePharma, China), RAW264.7 cells underwent treatment with this siRNA and were incubated for 48 h. Following 48‐h incubation, the culture media were replaced, and LPS at 1 µg/mL and GEN at 50 µM was added to their respective wells for a 24‐h incubation. After 24 h, the cells were collected for further analyses, including Western blotting and qRT‐PCR.

### 2.16. Statistical Analysis

GraphPad Prism edition 10 was used to analyze the data. Results were all displayed as mean ± SD. To compare several groups, one‐way ANOVA and the Student’s *t*‐test were used, employing *p*  < 0.05 as the significance threshold.

## 3. Results

### 3.1. GEN Alleviates the Symptoms of Colitis in Mice

**Figure 1:**
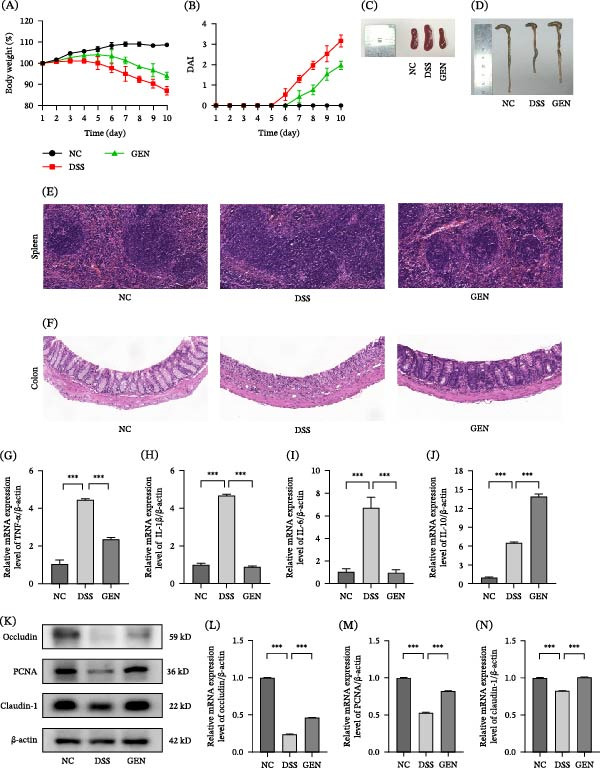
GEN alleviates DSS‐induced IBD in mice. (A) Percentage weight change in mice; (B) DAI score; (C) macroscopic appearance of the spleen; (D) macroscopic appearance of the colon; (E) H&E staining of the spleen (200×); (F) H&E staining of the colon (200×); (G–J) qRT‐PCR detection of inflammatory cytokine expressions (TNF‐α, IL‐1β, IL‐6, and IL‐10) in colon tissues; (K) Western blot detection of PCNA, occludin, and claudin‐1 levels in the colonic tissues; and (L–N) relative protein levels of occludin, PCNA, and claudin‐1 in the colon tissue employing ImageJ.  ^∗^ *p* < 0.05,  ^∗∗^ *p* < 0.01, and  ^∗∗∗^ *p* < 0.001.

The research model included mice randomly assigned to three categories: a negative control (*n* = 5), an IBD model triggered by DSS (*n* = 5), and a therapy group (DSS‐induced injury + GEN) (*n* = 5). Following colitis induction, weight assessment showed that although the DSS‐treated mice experienced a decrease in mean body weight, the GEN group’s body weight increased compared with the DSS group at day 10 (Figure 1A). The GEN group exhibited a lower mean DAI in contrast to the DSS group (Figure 1B). In addition, GEN therapy improved the gross morphological features of the mice’s spleen and colon tissues (i.e., reduced splenomegaly and increased colon length) (Figure 1C,D). Microscopically, H&E staining showed that GEN therapy preserved splenic and colonic structures better than the DSS group. Additionally, GEN therapy showed less crypt structural damage than the DSS group (Figure 1E,F). Analysis of cytokine expressions using qRT‐PCR revealed that GEN elevated the anti‐inflammatory cytokine IL‐10 while reducing the proinflammatory cytokines TNF‐α, IL‐1β, and IL‐6 (Figure 1G–J). To further determine the effect of GEN on colon tissue repair, it was revealed that the GEN therapy elevated the occludin, claudin‐1, and PCNA protein expression in the mouse colon more than the DSS group (Figure 1K). Additionally, ImageJ provided quantitative confirmation of the protein expression levels found in the blots (Figure 1L–N). As a result, our findings show that GEN alleviated colitis by improving weight, DAI, and colon’s macroscopic and microscopic morphology; reducing inflammation; and enhancing tight junction proteins.

### 3.2. GEN Improves the General Community Structure and Diversity of Gut Microbiota

**Figure 2:**
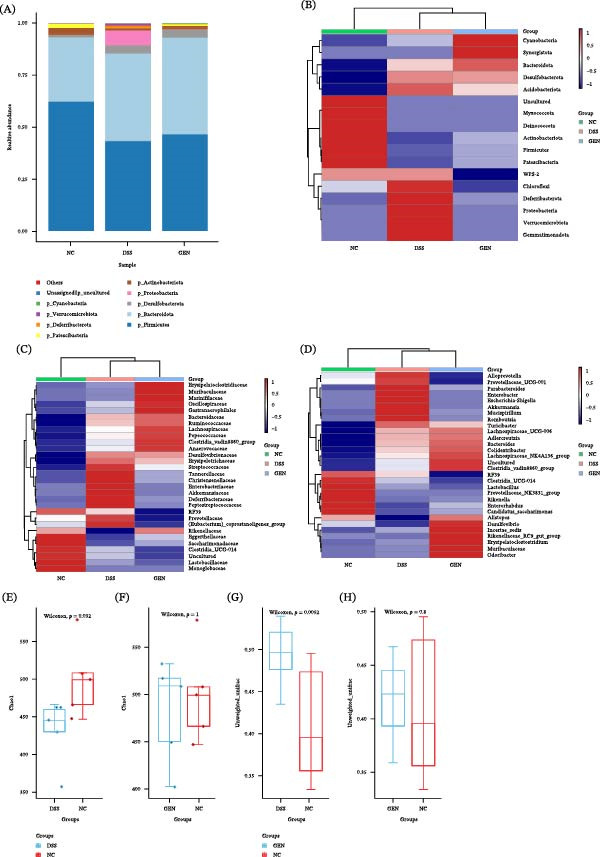
GEN improves the general community structure and diversity of gut microbiota. (A) Histogram of community abundance of the first 10 phyla of each group; (B) a cluster heat map of species abundance at the level of each group; (C) a cluster heat map of species abundance at the family level; (D) a cluster heat map of species abundance at the general level; (E) box plot of a diversity difference between groups based on the Chao1 index (NC vs. DSS group); (F) box maps of intergroup diversity differences using the Chao1 index (NC vs. GEN); (G) analysis of intergroup differentials in β diversity according to weighted UniFrac distance (group NC vs. DSS); and (H) analysis of intergroup differentials in β diversity following weighted UniFrac distance (group NC vs. GEN).

The effects of the therapy on the microbial composition and diversity were analyzed using 16S rDNA gene sequencing of fecal samples from mice. Quality control (QC) checks were used to assess species richness, distribution, and adequacy of sequencing data (Supporting Information 1: Figure S1A,B). Additionally, our results revealed that the abundance of the phyla *Firmicutes* and *Actinobacteriota* decreased, while the abundance of the phyla *Bacteroidota* and *Proteobacteria* increased in the DSS group; however, GEN increased the abundance of *Firmicutes*, *Bacteroidota*, and *Actinobacteriota* while decreasing the abundance of *Proteobacteria* (Figure 2A). A cluster heat map of species abundance at the level of each group also identified other phyla that increased in abundance in the DSS group, such as *Chloroflexi*, *Deferribacterota*, *Verrucomicrobiota*, and *Gemmatimonadota*, and were reversed by GEN (Figure 2B). At the family level, GEN reversed the increased abundance of *Streptococcaceae*, *Tannerellaceae*, *Christensenellaceae*, *Enterobacteriaceae*, *Akkermansiaceae*, *Deferribacteraceae*, and *Peptostreptococcaceae* in the DSS group. GEN increased the number of *Lachnospiraceae*, *Marinifilaceae*, *Oscillospiraceae*, *Peptococcaceae*, and *Rikenellaceae* at the family level (Figure 2C). A cluster heat map depicting species abundance at the general level revealed increased abundance of *Alloprevotella*, *Prevotellaceae_UCG-001*, *Parabacteroides*, *Enterobacter*, *Escherichia-Shigella*, *Akkermansia*, *Mucispirillum*, and *Romboutsia* in the DSS group, with these alterations being reversed by GEN. GEN additionally increased *Alistipes*, *Rikenellaceae_RC9_gut group*, and *Incerta_Sedis* at the general level (Figure 2D). There was a significant decrease in the alpha‐diversity index in the DSS group compared to the NC group (NC vs. DSS group, *p*‐value = 0.032) (Figure 2E) but no significant difference in the alpha‐diversity index in the GEN and NC groups (NC vs. GEN, *p*‐value = 1), although the GEN group had an increase in the alpha‐diversity index (Figure 2F). Additionally, there was a significant increase in the β diversity index in the DSS group compared to the NC group (NC vs. DSS *p*‐value = 0.0052) (Figure 2G) but no discernible difference between the NC and GEN groups (NC vs. GEN *p*‐value = 0.8) (Figure 2H). These data indicate that GEN can improve the diversity and community structure of the gut microbiota during colitis.

### 3.3. GEN Regulates Specific Bacterial Species, Restores Dysregulation, and Modulates Vital Functional Processes

**Figure 3:**
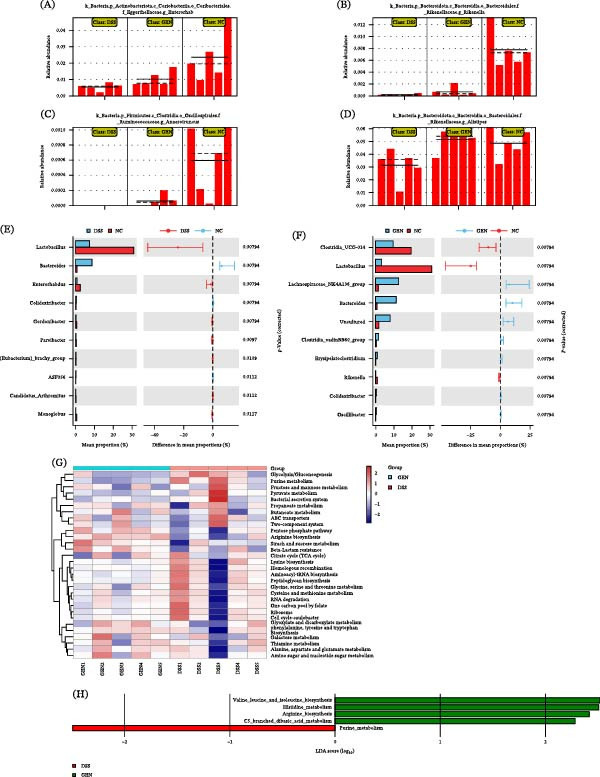
GEN regulates specific bacterial species and corrects dysregulation. (A–D) Comparison of microbial marker abundance among NC, DSS, and GEN categories (*Enterorhabdus*, *Rikenella*, *Anaerotruncus*, and *Alistipes*); (E) STAMP *t*‐test for species having significant differences between NC and DSS groups at the genus level; (F) STAMP *t*‐test of species between NC and GEN groups at the genus level; (G) KEGG heatmap prediction of bacterial function differences between DSS and GEN groups; and (H) KEGG LDA scores for anticipated functions in DSS and GEN groups.

Statistically distinct microbial communities and bacterial marker abundances within each group were identified using differential analyses. Differentially abundant species in various groups at different LDA scores are shown (Supporting Information 1: Figure S1C). GEN therapy restored underpopulated genera, including *Enterorhabdus*, *Rikenella*, *Anaerotruncus*, and *Alistipes*, compared to the DSS group (Figure 3A–D). Interestingly, the genera *Enterorhabdus*, *Rikenella*, *Anaerotruncus*, and *Alistipes* were the most differentially abundant bacteria among the NC and GEN groups (LDA score [log10] > 2) (Supporting Information 1: Figure S1C). This suggests that GEN regulates and restores specific bacterial genera to their normal levels. The STAMP differential analysis identified several bacterial communities that significantly differentiate the DSS group from the NC at the genus level. In particular, there was a decrease in the abundance of *Lactobacillus*, *Enterorhabdus*, *Gordonibacter*, *Parvibacter*, and *Candidatus_Arthromitus*, while the abundance of *Bacteroides*, *Colidextribacter*, and *ASF356* increased (Figure 3E). Moreover, STAMP differential analysis revealed several bacterial communities that significantly differentiate the GEN group from the NC group at the genus level, including increased *Lachnospiraceae_NK44A136_group*, *Clostridia vadinBB60group*, *Erysipelatoclostridium*, and *Oscillibacter*, as well as decreased *Clostridia_UCG_014* and *Lactobacillus* (Figure 3F). The KEGG heatmap prediction of bacterial function differences between DSS and GEN groups showed elevated purine metabolism in the DSS group, while arginine biosynthesis was elevated in the GEN group (Figure 3G). Other predicted functional items between the groups are found in Figure 3G. Further analysis using the KEGG LDA scores of anticipated functions across DSS and GEN categories revealed that the GEN group had higher arginine_biosynthesis and other predicted functions, in contrast to the DSS category, which had higher purine_metabolism (Figure 3H). Thus, GEN regulated specific bacterial species, restored dysregulation, and modulated vital functional processes in colitis mitigation.

### 3.4. GEN Reduces Dysregulation of Intestinal Metabolites in Alleviating Colitis

**Figure 4:**
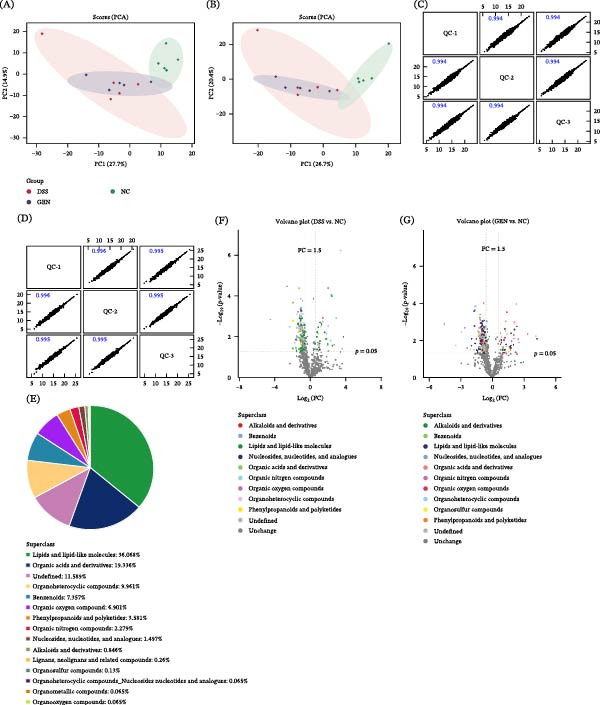
GEN reduces dysregulation of intestinal metabolites in alleviating colitis. (A) PCA analysis of the total sample in positive ion mode; (B) PCA analysis of the total samples in negative ion mode; (C) correlation map of QC samples in positive ion mode; (D) correlation map of QC samples in negative ion mode; (E) percentage of metabolites identified in each chemical class; (F) volcano plot showing differentially expressed metabolites between the NC and DSS groups in positive ion mode; and (G) volcano plot showing differentially expressed metabolites between the NC and GEN groups in positive ion mode.

Samples from positive and negative ion modes were QC using the overlapping spectra of total ion current maps (Supporting Information 1: Figure S1D,E). To ascertain the cluster distribution of metabolites within each group, the NC exhibited a greater clustering, distinctly separated from the GEN and DSS groups. However, the GEN group had metabolites clustered within the DSS group in positive and negative modes (Figure 4A,B). The correlation map for QC samples in positive mode revealed a positive link among them (Figure 4C). A similar correlation map trend was found for the QC samples in the negative mode (Figure 4D). The total number of metabolites discovered was derived from 14 superclass chemical classifications (Figure 4E), and the volcano plot revealed an upregulation and downregulation of differentially expressed metabolites between the groups (Figure 4F,G). Multivariate analysis further revealed that GEN altered metabolites dysregulated in the DSS group (Supporting Information 1: Figure S1F,G). Thus, GEN controlled dysregulated metabolites in DSS to alleviate colitis.

### 3.5. GEN Regulates the Arginine Metabolic Pathway to Reduce Colitis

To assess whether GEN regulates the arginine metabolic pathway to reduce IBD, a functional analysis of differentially expressed metabolites and associated pathways was performed. Hierarchical cluster analysis of intragroup expression changes in the KEGG positive mode indicated differential expression of metabolites among the NC, DSS, and GEN groups (Figure 5A). A similar differential expression of metabolites in negative mode was also observed among the groups (Figure 5B). Metabolites with significant differences in both ion modes showed distinct clusters among the three groups (Supporting Information 1: Figure S1H,I). A multiplex analysis of metabolite levels in the DSS and GEN groups in positive ion mode revealed that G‐guanidinobutyrate and other metabolites were elevated (Figure 5C). Additionally, a differential metabolite graph of the network revealed the interaction of G‐guanidinobutyrate between three defined superclasses of molecules between the GEN and DSS groups, including lipids and lipid‐like molecules, organic acids and derivatives, and organoheterocyclic compounds (Figure 5D). Therefore, to identify which group upregulated G‐guanidinobutyrate, cluster heat maps of differential metabolites in the KEGG pathway showed that GEN increased the expression of G‐guanidinobutyrate and other metabolites; however, the DSS group decreased their expression (Figure 5E). Figure 5F depicts the detailed relationship between G‐guanidinobutyrate and other metabolites involved in arginine and proline metabolic processes. Thus, GEN regulates the arginine metabolic pathway (G‐guanidinobutyrate‐mediated) to reduce colitis.

Figure 5GEN regulates the arginine metabolic pathway to reduce colitis. (A) Hierarchical cluster analysis of intragroup expression changes in KEGG positive mode; (B) hierarchical cluster analysis of intragroup expression changes under KEGG negative ion mode; (C) multiplex analysis of significant differences in metabolite expression between DSS and GEN groups in positive ion mode; (D) differential metabolite graph of a network in DSS and GEN groups; (E) cluster heat maps of different metabolites in KEGG pathway of DSS and GEN groups; and (F) KEGG pathway map for the arginine and proline metabolic process, including differential metabolites.

**Figure:**
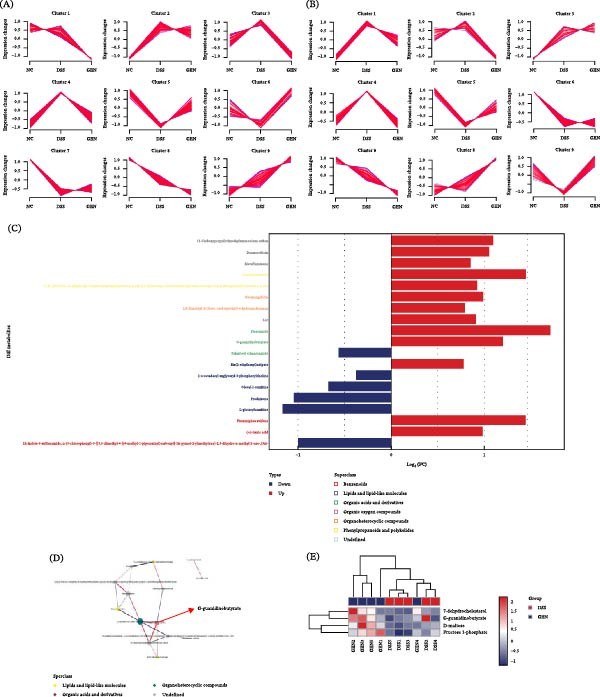

**Figure:**
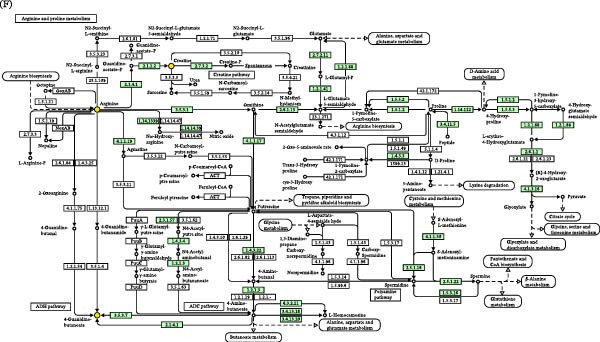

### 3.6. The Microbiome and Arginine Metabolism Regulation by GEN During Remission of Colitis

**Figure 6:**
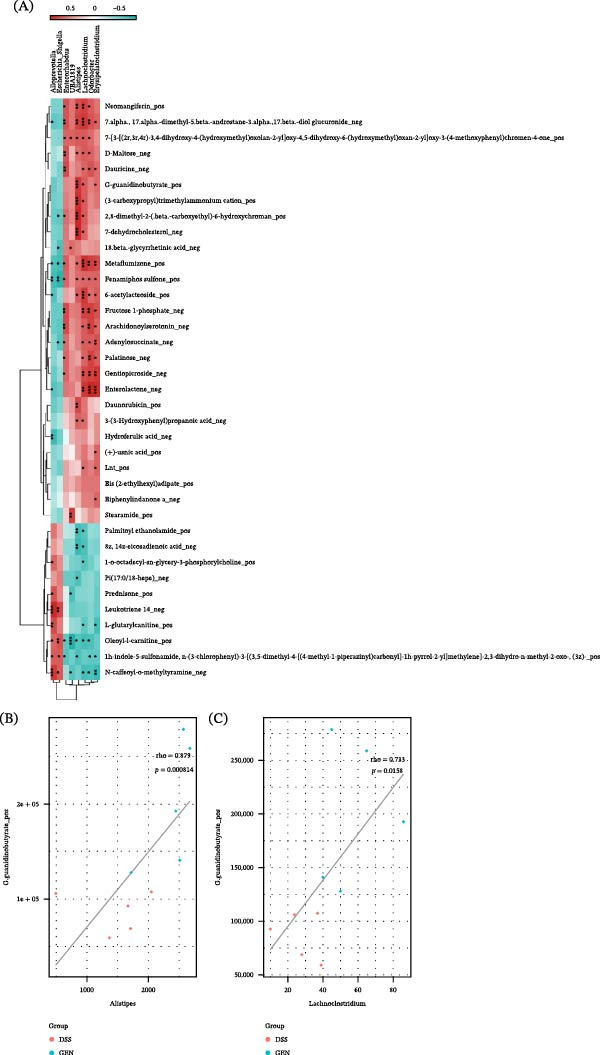
The regulation of the microbiome‐arginine metabolism axis by GEN during the remission phase of colitis. (A) Spearman correlation analysis of significant difference bacteria and significant difference metabolites. (B, C) Scatter diagram of correlation.

A heat map of the Spearman correlation coefficient matrix and a network analysis of the Spearman correlation between these gut microbiota and metabolites were constructed (Supporting Information 1: Figure S1J,K). Spearman correlation analysis showed that between the GEN and DSS groups, eight bacterial genera (*Alloprevotella*, *Escherichia-Shigella*, *Enterorhabdus*, *UBA1819*, *Alistipes*, *Lachnoclostridium*, *Odoribacter*, and *Erysipelatoclostridium*) and 37 metabolites were correlated (Figure 6A). It was further shown that G‐guanidinobutyrate significantly correlated with the bacterial genera *Alistipes* and *Lachnoclostridium*, and a scatter diagram showed a positive correlation between these genera and G‐guanidinobutyrate (Figures 6B,C). These findings suggest that the gut microbiota may regulate arginine metabolism during the remission phase of colitis.

### 3.7. GEN Regulates Colon Mucosal Arginine Metabolism in Mice to Reduce Colitis

**Figure 7:**
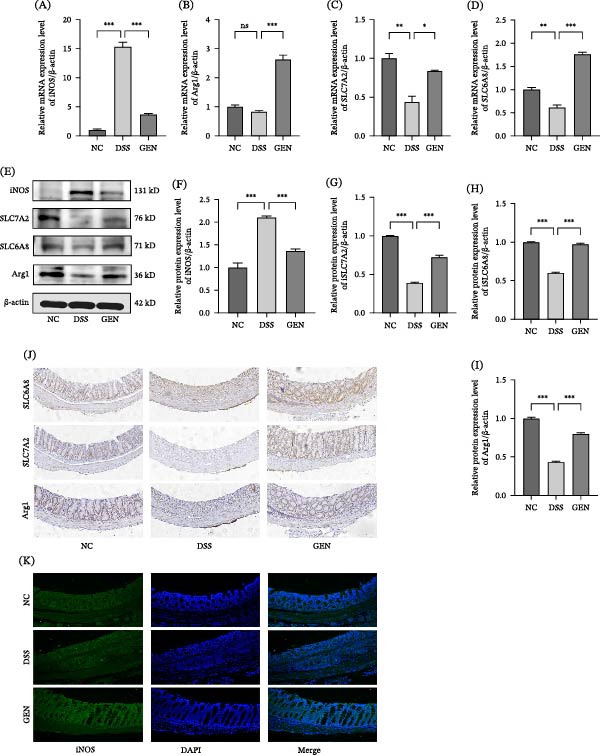
GEN regulates arginine metabolism in the colonic mucosa of mice to reduce colitis. (A–D) qRT‐PCR analysis of transcript levels of molecules associated with arginine metabolism (iNOS, SLC7A2, SLC6A8, and Arg1) in mouse colonic tissue; (E) Western blot study of arginine metabolic molecule protein expression levels in the colon of mice; (F–I) relative protein expression levels of iNOS, SLC7A2, SLC6A8, and Arg1 in the mouse colon tissues using ImageJ; (J) the expression levels of SLC7A2, SLC6A8, and Arg1 analyzed by IHC (200×); and (K) the expression level of iNOS (200×) in the mouse colon was analyzed by IF.  ^∗^ *p* < 0.05,  ^∗∗^ *p* < 0.01, and  ^∗∗∗^ *p* < 0.001.

After discovering that GEN regulates arginine metabolism via G‐guanidinobutyrate, we investigated its role in modulating arginine metabolism in the colon mucosa to reduce colitis in mice. Following the initiation of the inflammatory model, qRT‐PCR showed that GEN significantly reduced the mRNA expressions of iNOS and increased the mRNA expressions of SLC7A2, SLC6A8, and Arg1 relative to the DSS group (Figure 7A–D). Comparable outcomes were noted in the Western blot analysis of arginine metabolic proteins (Figure 7E), with ImageJ utilized for quantifying protein expression levels (Figure 7F–I). To further investigate the expression levels of molecules related to arginine metabolism at both the tissue and cellular levels, IHC and IF analyses were performed. The IHC results indicated that GEN restored the expression of SLC7A2, SLC6A8, and Arg1 relative to the DSS group (Figure 7J). In contrast, IF analysis revealed lower iNOS fluorescence intensity in the GEN group than in the DSS group (Figure 7K). These findings suggest that GEN may modulate arginine metabolism–related molecules, contributing to the reduction of colitis.

### 3.8. GEN Reduces the Toxicity of Macrophages, Modulates Cytokines in Macrophages to Reduce Inflammation, and Regulates Changes in the Cell Cycle in Macrophages

**Figure 8:**
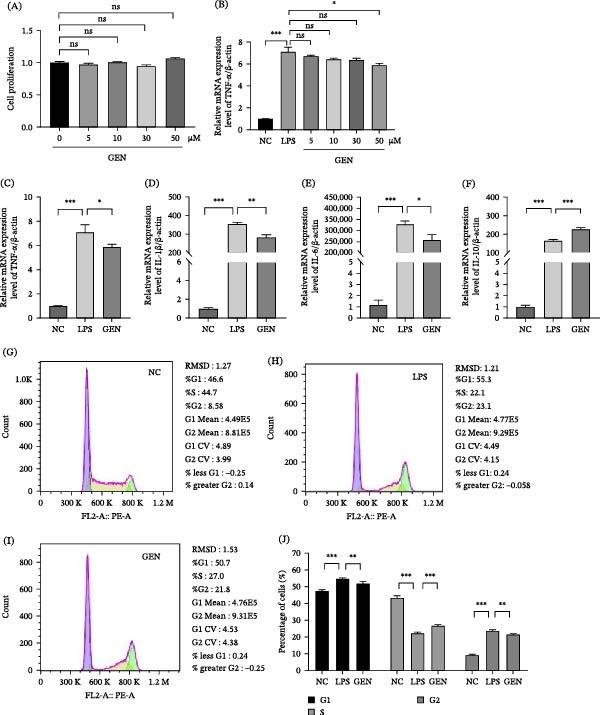
GEN reduces the toxicity of macrophages, modulates cytokines in macrophages to reduce inflammation, and regulates changes in the cell cycle in macrophages. (A) CCK8 detection of the impact of various GEN dosages on the RAW264.7 cell proliferation; (B) qRT‐PCR analysis of the impact of various GEN dosages on cell inflammation induced by LPS; (C–F) qRT‐PCR analysis of the expression levels of inflammatory factors (TNF‐α, IL‐1β, IL‐6, and IL‐10) mRNA in macrophages RAW264.7; and (G–J) detection of changes in the cell cycle of GEN‐treated RAW264.7 cells.  ^∗^ *p* < 0.05,  ^∗∗^ *p* < 0.01, and  ^∗∗∗^ *p* < 0.001.

To determine whether GEN treatment was nontoxic to cell proliferation, CCK8 assays showed that GEN at concentrations up to 50 µM was not toxic (Figure 8A). Additionally, qRT‐PCR revealed a substantial decrease in TNF‐α expression at 50 µM in the GEN group compared to the LPS group (Figure 8B). Thus, 50 µM was used for subsequent experiments. Cytokine expression analysis was performed using qRT‐PCR, and the results indicated that GEN significantly decreased the relative inflammatory cytokine (TNF‐α, IL‐1β, and IL‐6) mRNA expressions while increasing the levels of the anti‐inflammatory cytokine IL‐10 (Figure 8C–F). Cells typically enter the S stage (DNA synthesis) after passing through the G1/S checkpoint. Imbalanced cell cycle regulatory proteins lead to cellular arrest in the G1 phase, evidenced by an increased percentage of cells in G1 (attributable to checkpoint inhibition and accumulation) and a reduced percentage in the S phase (due to inadequate DNA synthesis and reduced proliferative capacity) [31]. Hence, we sought to determine whether GEN could maintain normal cell cycle processes in macrophages. It was found that GEN significantly reduced the cell proportions in the G1 or G2 stages and significantly increased the S phase (Figure 8G–J) compared to the LPS group, implying that GEN could enhance the proliferation of cells and control the normal cell cycle. Thus, these results indicate that GEN at 50 µM reduces macrophage toxicity, modulates cytokine expression, and regulates cell cycle changes in macrophages.

### 3.9. GEN Regulates the Arginine Metabolic Pathway of Macrophages to Reduce Inflammation

**Figure 9:**
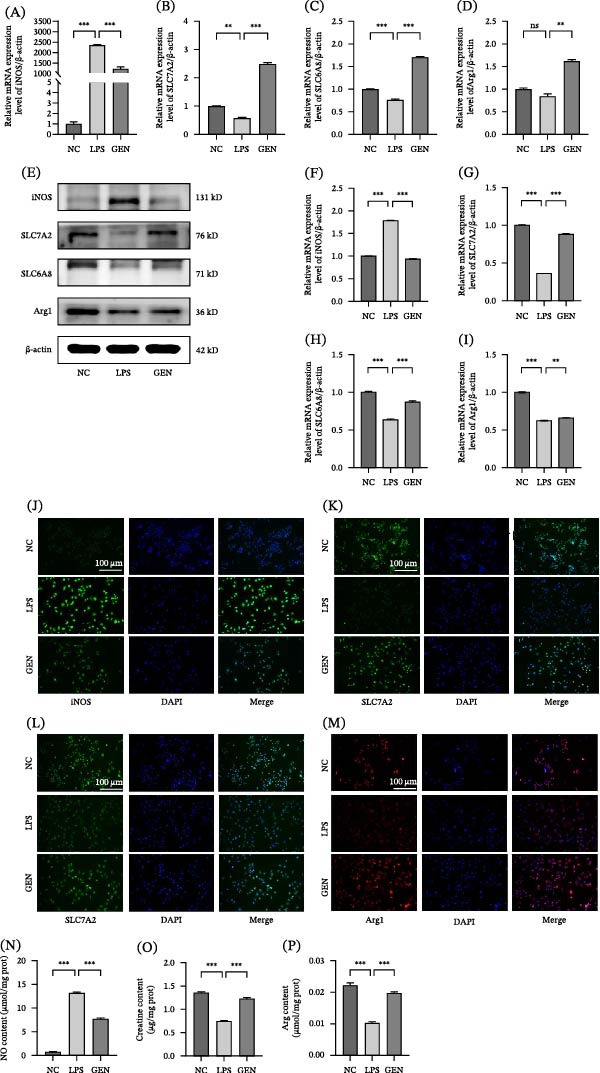
GEN controls arginine metabolism–related molecules in macrophages. (A–D) qRT‐PCR examination of the mRNA expression levels of markers associated with arginine metabolism (iNOS, SLC7A2, SLC6A8, and Arg1) in RAW264.7 macrophages; (E) Western blot study of RAW2647 cells’ protein expression levels of molecules linked to arginine metabolism; (F–I) relative protein expression levels of iNOS, SLC7A2, SLC6A8, and Arg1 in RAW264.7 cells using ImageJ; (J–M) IF analysis of the expression levels of molecules associated with arginine metabolism in RAW264.7 macrophages; and (N–P) detection of macrophage NO, creatine, and arginine content by micromethod.  ^∗^ *p* < 0.05,  ^∗∗^ *p* < 0.01, and  ^∗∗∗^ *p* < 0.001.

To determine the effect of GEN on arginine metabolism–related molecules (iNOS, SLC7A2, SLC6A8, and Arg1) in macrophages, qRT‐PCR was done, and results showed that GEN decreased the relative iNOS mRNA expression and elevated the mRNA expression of SLC7A2, SLC6A8, and Arg1 in macrophages (Figure 9A–D). Similar results were also found in the Western blot analysis (Figure 9E), which aligned with findings from the colitis mouse model. ImageJ was used to further confirm the protein expression of the Western blot bands (Figure 9F–I). To assess the expression of the arginine metabolism at the cellular level, IF was performed. IF showed increased fluorescence intensity of SLC7A2, SLC6A8, and Arg1 and decreased iNOS in the GEN‐treated group compared to the LPS‐treated group (Figure 9J–M). Other molecules in the arginine metabolic pathway were observed using microplate detection, which showed increased NO and decreased creatine and Arg levels in macrophages treated with LPS; however, GEN treatment reversed all these changes (Figure 9N–P). Thus, GEN regulated macrophage‐arginine metabolism to reduce inflammation.

### 3.10. Analysis of Drug Target Sequencing Results and Expression Levels of Anxa5 and COX‐2 in RAW264.7 Cells and Mouse Models

**Figure 10:**
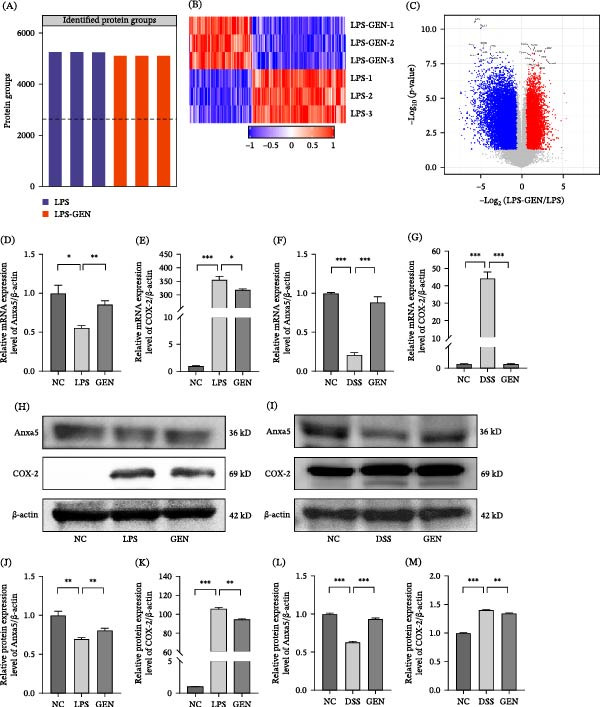
Analysis of drug target sequencing results and expression levels of Anxa5 and COX‐2 in mouse models and RAW264.7 cells. (A) Statistical histogram of protein identification and quantification results; (B) cluster analysis among detected proteins; (C) volcano plot showing peptide segments with significant differences in expression; (D, E) qRT‐PCR detection of the transcript levels of Anxa5 and COX‐2 in RAW264.7 cells; (F, G) qRT‐PCR detection of the transcript levels of Anxa5 and COX‐2 in mouse; (H) Western blot detection of the expression levels of Anxa5 and COX‐2 in RAW264.7 cells; (I) Western blot detection of the expression levels of Anxa5 and COX‐2 in mouse; (J, K) relative protein expression levels of COX‐2 and Anxa5 using ImageJ; and (L, M) relative protein expression levels of COX‐2 and Anxa5 using ImageJ.  ^∗∗∗^ *p* < 0.001,  ^∗∗^ *p* < 0.01, and  ^∗^ *p* < 0.05.

To determine the protein targeted by the GEN and LPS treatments, drug target sequencing was performed. A statistical histogram of protein identification and quantification results was generated (Figure 10A), and a cluster analysis of discovered proteins was performed (Figure 10B). A volcano plot demonstrated upregulation and downregulation of selected proteins affected by both LPS and GEN treatments (Figure 10C). It was further found that the GEN therapy (GEN + LPS) targeted Anxa5 (upregulation) (Figure 10C). After confirming that GEN targeted Anxa5, we assessed whether GEN therapy elevated Anxa5 levels and influenced COX‐2 expression in a RAW264.7 cell model. The mRNA levels of Anxa5 and COX‐2 were measured using qRT‐PCR. The results showed that Anxa5 mRNA expression was significantly higher than in the LPS treatment group (Figure 10D), although COX‐2 mRNA expression was suppressed (Figure 10E) following GEN treatment. Similar results were observed during Western blotting (Figure 10H). To further confirm Western blot protein expression, ImageJ served to quantify the blot bands (Figure 10J,K). Additionally, a mouse model was developed to determine whether GEN treatment increased Anxa5 levels and affected COX‐2 expression, comparable to the RAW264.7 cell model. The Anxa5 and COX‐2 mRNA levels were evaluated by qRT‐PCR, yielding similar outcomes (Figure 10F,G), consistent with findings from the RAW264.7 cell model. Furthermore, Western blot analysis yielded comparable results (Figure 10I), and relative protein expressions were quantified using ImageJ (Figure 10L,M). This suggests that during GEN therapy, Anxa5 levels increase, and COX‐2 levels decrease. Therefore, Anxa5 could be considered a potential therapeutic target for GEN treatment.

### 3.11. GEN Regulates the NF‐κB Pathway–Related Proteins in the RAW264.7 Cell and Animal Model to Alleviate Colitis

**Figure 11:**
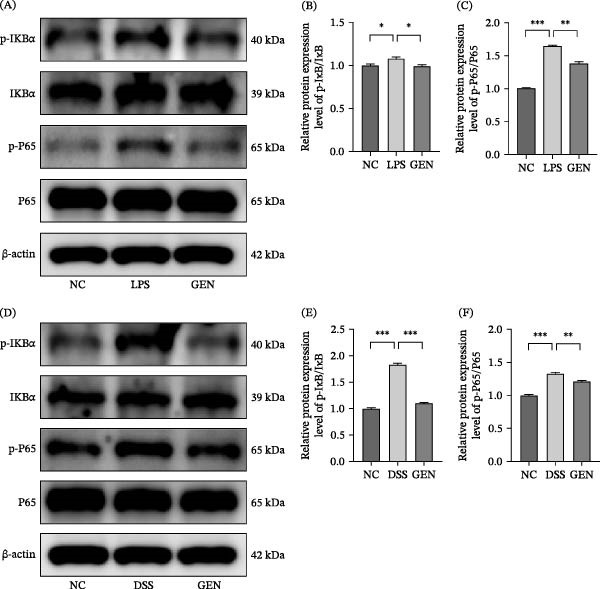
Regulation of the NF‐κB pathway–related proteins in RAW264.7 cells and a mouse model by GEN to alleviate colitis. (A, D) Western blot detection of NF‐κB pathway–related proteins (p‐IkBα, IkBα, p‐P65, and P65) expression levels in RAW264.7 cells and mouse colonic tissues. (B, C, E, F) ImageJ analysis of the relative protein expression of p‐IkBα/IkBα and p‐P65/P65.  ^∗∗∗^ *p* < 0.001,  ^∗∗^ *p* < 0.01, and  ^∗^ *p* < 0.05.

Following confirmation that GEN targets Anxa5 to downregulate COX‐2 expression, we determined whether GEN could regulate the NF‐κB pathway–related proteins in macrophages to alleviate colitis. Western blot analysis revealed that the LPS group increased the expression of p‐IkBα and p‐P65; however, administration of GEN reduced these levels (Figure 11A). Additionally, ImageJ quantitatively confirmed these expression levels (Figure 11B,C). Similar findings were also observed in the mouse model of colitis (Figure 11D–F), which was consistent with the RAW264.7 cell model. P65 and IkBα expression levels were unchanged in both models, suggesting that the phosphorylated versions of the NF‐κB pathway are implicated in colitis. Hence, GEN may target these phosphorylated forms in the colon to reduce inflammation.

### 3.12. Expression of COX‐2, Inflammatory Factors, and the NF‐κB Pathway–Related Proteins After RAW264.7 Cells Were Transfected With Anxa5 siRNA

**Figure 12:**
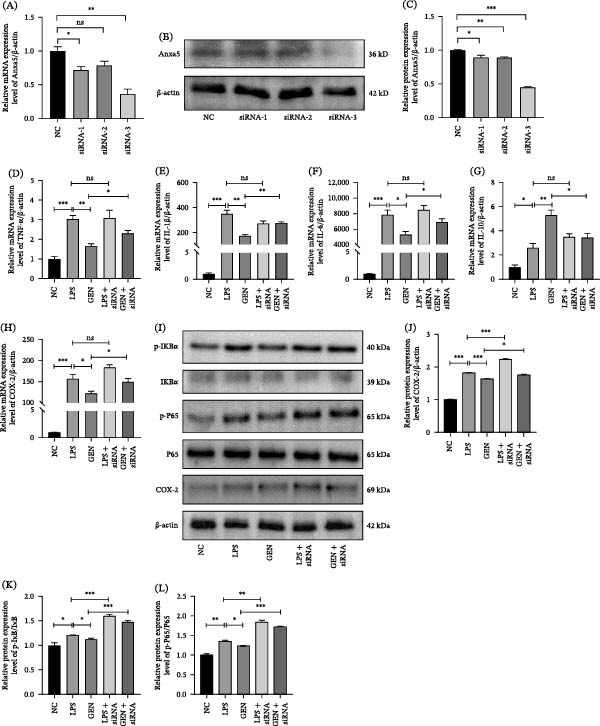
(A) qRT‐PCR detection of the efficiency of siRNA‐mediated knockdown of Anxa5 at the transcriptional level; (B) Western blot to detect the efficiency of siRNA knockdown of Anxa5 at a translational level; (C) relative protein expression of siRNA knockdown of Anxa5; (D–H) qRT‐PCR detection of the transcriptional levels of COX2 and inflammatory cytokines in RAW264.7 cells; (I) Western blot detection of COX2 and NF‐κB pathway–related proteins expression levels in RAW264.7 cells; and (J–L) relative protein expression of NF‐κB pathway–related proteins and COX‐2 using ImageJ.  ^∗∗∗^ *p* < 0.001,  ^∗∗^ *p* < 0.01, and  ^∗^ *p* < 0.05.

After confirming that siRNA‐3 successfully knocked down Anxa5 in our cell model using qRT‐PCR (Figure 12A), Western blot (Figure 12B), and ImageJ (Figure 12C), we aimed to investigate whether GEN therapy, which has previously been demonstrated to target Anxa5 for its anti‐inflammatory properties in reducing colitis, could effectively target Anxa5 when used in conjunction with siRNA‐3. As a result, qRT‐PCR detection was performed, demonstrating that GEN + siRNA significantly raised COX‐2 (Figure 12H) and proinflammatory cytokines (Figure 12D–F) while decreasing anti‐inflammatory cytokine (Figure 12G) mRNA expressions compared to GEN alone. These findings suggest that knocking down Anxa5 may reduce GEN activity. Hence, GEN may not efficiently target Anxa5, increasing COX‐2 and proinflammatory cytokines while lowering anti‐inflammatory cytokines. To further confirm the expression of proteins linked to the NF‐κB pathway and COX‐2 after RAW264.7 cell transfection with Anxa5 siRNA‐3, following qRT‐PCR detection, Western blotting was done. It was revealed that the COX‐2, p‐IkBα, and p‐P65 protein expression levels were higher in the GEN + siRNA group than in the GEN group (Figure 12I). Similar observations were also seen in the LPS + siRNA and LPS groups (Figure 12I). However, IkBα and P65 remained relatively unchanged. ImageJ analysis further confirmed these expression levels quantitatively (Figures 12J–L). These findings suggest that GEN may modulate COX‐2, inflammatory cytokines, and proteins associated with the NF‐κB pathway to reduce colon inflammation by targeting Anxa5; hence, Anxa5 may be a therapeutic target for GEN.

## 4. Discussion

IBD is characterized by a cytokine imbalance that promotes and inhibits inflammation, leading to tissue damage and disease onset. A new treatment approach specifically targets these cytokines [33]. Additionally, the stability of the epithelial barrier depends on proteins involved in tight junctions, such as occludin, claudins, and zonula occludens; any imbalances in these proteins can compromise the barrier’s integrity. It is believed that the disintegration of the gut barrier contributes to IBD [34]. Our study revealed that GEN reduced DAI, increased anti‐inflammatory cytokines (IL‐10), decreased proinflammatory cytokines (TNF‐α, IL‐1β, and IL‐6), and increased tight junction proteins such as occludin and claudin‐1, suggesting increased epithelial barrier integrity to minimize colitis. The macroscopic and microscopic features were restored by GEN administration. Consistent with our findings, other studies have demonstrated that GEN reduces DAI, decreases proinflammatory cytokines, and alleviates both macroscopic and microscopic features of colitis while also increasing tight junction expression to prevent colitis [26, 30].

IBD has been linked to dysbiosis of the gut microbiota [35]. A comparative microbiota analysis of a specimen using 16S rDNA sequencing is sufficient to determine the relative amount of 16S rDNA and potentially harmful species [36]. The study used 16S rDNA sequencing to evaluate the gut microbiota community structure, diversity, and functions, with the goal of better understanding the role of GEN in these parameters. Our study found that GEN increased the abundance of *Firmicutes*, *Bacteroidota*, and *Actinobacteriota* and reduced *Proteobacteria*. Additionally, GEN decreased the abundance of *Alloprevotella*, *Enterobacter*, *Escherichia-Shigella*, and *Akkermansia*. The abundance of *Alloprevotella*, *Escherichia-Shigella*, *Enterobacter*, and *Akkermansia* is increased in IBD [37, 38], while *Firmicutes* are decreased [39]. *Proteobacteria* are more prevalent in IBD patients [40]. However, GEN reversed all these changes from our study. Moreover, GEN therapy helped restore underpopulated genera such as *Enterorhabdus*, *Rikenella*, *Anaerotruncus*, and *Alistipes*. Preclinical studies indicate that the gut bacteria’s alpha diversity is lower and more distinct in DSS than in controls [41, 42]; however, GEN restored gut microbiota diversity. KEGG LDA scores of predicted functions showed elevated valine_leucine_and_isoleucine_biosynthesis, histidine_metabolism, arginine_biosynthesis, and C5_branched_dibasic_acid_metabolism in the GEN group, whereas the DSS group exhibited increased purine_metabolism. Thus, this indicates that GEN regulated the gut microbiota community structure, diversity, and function.

Metabolites, small molecular particles produced by microbial metabolism, are vital communication channels between the gut microbiota and the host [7]. Changes in the composition and function of the microbiota, as well as the metabolites they produce, have been linked to several diseases, including IBD. Signals from these microorganisms influence immune balance, immune development, host energy breakdown, and preservation of mucosal stability [7, 43, 44]. According to our findings, GEN raised the expression of G‐guanidinobutyrate and other metabolites, whereas the DSS group lowered it. G‐guanidinobutyrate was shown to be involved in arginine metabolism. Arginine metabolism plays a significant role in the pathophysiology of IBD by primarily inhibiting inflammation through the arginine‐polyamine and arginine‐creatine axes, in contrast to the proinflammatory iNOS axis [13]. The metabolism of arginine produces creatine. Also, SLC7A2 and SLC6A8 are arginine and creatine transporters, respectively. The increased iNOS activity in inflammatory tissue during IBD flare‐ups boosts luminal NO production [45]. SLC6A8 (CRT) knockout organoids have shown impaired barrier formation [46]. Moreover, SLC7A2‐deficient mice are more susceptible to DSS‐induced colitis [47]. Mice exposed to DSS also exhibit a reduction in Arg1 [48]. Therefore, in the colon mucosa, GEN decreased iNOS expression while increasing SLC7A2, SLC6A8, and Arg1 (arginine metabolism–related molecules). Thus, GEN may regulate the arginine metabolic pathway in the colon mucosa, thereby preventing colitis.

Macrophages play a role in maintaining intestinal immunological homeostasis, regulating tissue healing, and inducing inflammatory remission. Macrophage polarization leads to major alterations in Arg metabolism, with iNOS and Arg1 enzymes being the key players. Arg also generates creatine, a crucial cellular energy source [14, 49]. The results of our study indicated that GEN reduced the relative expression of inflammatory cytokines (IL‐1β, TNF‐α, and IL‐6) and raised IL‐10, an anti‐inflammatory cytokine, levels in macrophages. It is acknowledged that macrophages can be polarized into an M2 anti‐inflammatory phenotype that can synthesize IL‐10. This suggests that GEN may have polarized the M2 phenotype to prevent inflammation. Immunoregulatory M2 macrophages that produce IL‐10 are characterized by Arg1, the enzyme that catalyzes the conversion of arginine to ornithine [50]. Conversely, iNOS, a crucial enzyme in the macrophage inflammatory response, produces NO and is strongly triggered by proinflammatory stimuli [51]. Also, creatine alters macrophage polarization by increasing IL‐4‐STAT6–activated Arg1 expression and inhibiting immunological effector molecules, encompassing IFN‐γ‐JAK‐STAT1 transcription factor signaling and iNOS. The reduced expression of SLC6A8 promotes intracellular creatine depletion, which affects immune responses mediated by macrophages [14]. SLC7A2 transports arginine, and the high‐affinity transporter SLC7A2B is broadly distributed in macrophages [15, 16]. This study found that GEN decreased iNOS and NO levels in macrophages while boosting the expression of SLC7A2, SLC6A8, Arg1, creatine, and Arg. These findings suggest that GEN can regulate the macrophage‐arginine axis to prevent inflammation.

Collectively, GEN therapy restored underpopulated genera, including *Enterorhabdus*, *Rikenella*, *Anaerotruncus*, and *Alistipes*, compared to the DSS group, and these bacterial markers were the most differentially abundant between the NC and GEN groups. Furthermore, *Alistipes* was shown to significantly correlate (positively) with the metabolite G‐guanidinobutyrate, where G‐guanidinobutyrate was shown to be involved in the arginine and proline metabolism processes. After discovering that GEN could regulate arginine metabolism via G‐guanidinobutyrate by elevating *Alistipes* levels, we investigated the role of GEN in modulating arginine metabolism in macrophages, and it was found that GEN reduced colitis by increasing the expression of arginine metabolism–related molecules SLC7A2, SLC6A8, Arg1, creatine, and Arg while decreasing iNOS and NO levels. This defines the gut microbiota‐macrophage‐arginine metabolism process. Comparable results were noted in mouse models of colitis, wherein GEN elevated the expression levels of SLC7A2, SLC6A8, and Arg1 while diminishing iNOS to alleviate colitis. Therefore, these show that GEN modulation of specific gut microbiota led to arginine metabolism via the production of the metabolite G‐guanidinobutyrate. This led to the reduction of inflammation by modulating the arginine metabolism–related molecules in both macrophages and the colon. This confirms that the regulation of the gut bacteria was associated with the resolution of inflammation.

The NF‐κB family consists of dimeric transcription factors [52]. Although disruption of the NF‐κB pathway is linked to IBD [52], Anxa5 has been shown to protect against it. Baek and colleagues found that Anxa5 overexpression reduced TNF‐α–stimulated COX‐2 expression. At the same time, Anxa5 knockdown reversed the suppression of COX‐2 production by auranofin, which might stimulate Anxa5 expression [19]. Additionally, in prostate cancer cells, Anxa5 knockdown results in phosphorylation of NF‐κB p65, suggesting that Anxa5 downregulates COX‐2 by preventing p65 activation [19]. Our study found that GEN therapy targeted Anxa5 while downregulating COX‐2. GEN downregulated proinflammatory cytokines and elevated anti‐inflammatory cytokines and inhibited the proteins associated with the NF‐κB pathway. However, knockdown of Anxa5 reduced the efficacy of GEN, leading to increased COX‐2 expression, increased proinflammatory cytokines, decreased anti‐inflammatory cytokines, and the activation of the NF‐κB pathway–related proteins.

**Figure 13:**
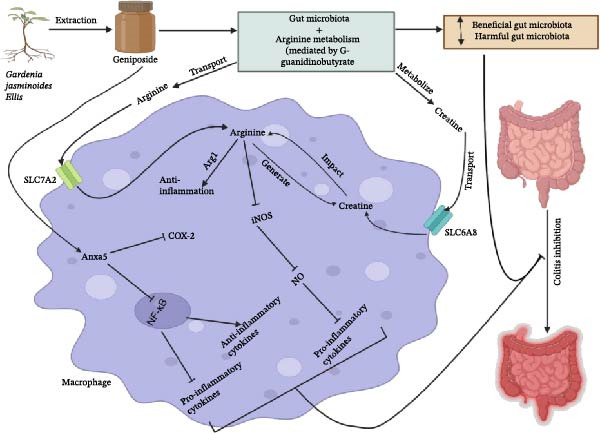
The GEN mechanism theory for reducing IBD. GEN increased arginine metabolism in macrophages by increasing beneficial gut microbes, SLC7A2, SLC6A8, creatine, Arg, and Arg1, and the downregulation of iNOS and NO. Additionally, GEN increased Anxa5, which mediates macrophage polarization, and downregulated the NF‐κB signaling, inhibiting colitis.

Mechanistically, GEN modulated the gut microbiota‐macrophage‐arginine metabolism and the Anxa5 axis, resulting in the resolution of colitis by preventing the production of proinflammatory cytokines (Figure 13).

Recently, therapies targeting IBD, such as corticosteroids, immunosuppressants, and biologics, have not yielded a positive response in patients and may lead to side effects. However, GEN, a natural component of traditional Chinese medicine that regulates the gut microbiota and arginine metabolism and inhibits the NF‐κB pathway by targeting Anxa5 to reduce IBD, may lessen the side effects of IBD medications. Additionally, GEN at 50 μM was demonstrated to be nontoxic to cells. Therefore, oral daily administration of GEN at a calculated human dose for a specified duration could potentially be used as an alternative or complementary naturopathic therapeutic option for patients with IBD‐associated colitis and reduce IBD medication costs as well.

Although GEN has been shown to alleviate colitis, the study indicated that GEN increased the levels of G‐guanidinobutyrate, which positively correlated with beneficial gut microbiota. However, the relationship between microorganisms and arginine metabolism–related molecules is not well understood, suggesting a need for further investigation.

## 5. Conclusion

GEN reduced colitis in mice by alleviating clinical symptoms, boosting tight junction proteins, enhancing the intestinal microbiota structure and diversity, and decreasing metabolite dysregulation. These outcomes were associated with arginine‐induced macrophage polarization via the upregulation of SLC7A2, SLC6A8, creatine, Arg, and Arg1, alongside the downregulation of iNOS and NO. Additionally, GEN increased Anxa5, which mediates macrophage polarization, and downregulated the NF‐κB pathway. This identifies the microbiota‐macrophage‐arginine metabolism and Anxa5 axis as potential therapeutic targets for intestinal inflammation.

**Nomenclature**
Anxa5: Annexin A5
Arg: L‐arginine
Arg1: Arginase 1
COX‐2: Cyclooxygenase‐2
DSS: Dextran sulfate sodium
GEN: Geniposide
IBD: Inflammatory bowel disease
IHC: Immunohistochemistry
IF: Immunofluorescence
iNOS: Inducible nitric oxide synthase
LPS: Lipopolysaccharide
NF‐κB: Nuclear factor kappa B
NO: Nitric oxide
SLC6A8: Solute carrier family 6 member 8
SLC7A2: Solute carrier family 7 member 2.

## Author Contributions

**Xiu Wang, Francis Atim Akanyibah, and Pengfei Zhang**: conception and design, collection and/or assembly of data, data analysis and interpretation, manuscript writing. **Xiaodong Zhou**: funding acquisition, collection and/or assembly of data and interpretation. **Ming Zhang**: collection and/or assembly of data and interpretation. **Fei Mao**: study design, data analysis and interpretation, and final approval of the manuscript.

## Funding

This work was supported by the Zhenjiang Key Research and Development Plan (Social Development) (Grant SH2024047), the Key Research and Development (Social Development) Projects of the Innovation Special Fund of Danyang (Grant SSF202410), the Henan Province 2024 Science and Technology Development Plan (Grant 242102310081), the Open Topic at the University Level of Shangqiu Medical College in 2023 (Grant KFKT23005), the Suqian Natural Science Fund Project (Grant K202423), the Key Project of Health Commission of Jiangsu Province (Grant K2024015), and the Jiangsu Provincial Medical Key Discipline Cultivation Unit (Grant JSDW202241).

## Disclosure

The ARRIVE guidelines 2.0 author checklist was utilized in the preparation and revision of the manuscript. The research guideline checklist is included as a Supporting Information 2. All authors read and approved the final manuscript.

## Ethics Statement

The animal experiment study received approval from Jiangsu University’s Ethical Committee (UJS‐IACUC‐2024062602).

## Conflicts of Interest

The authors declare no conflicts of interest.

## Supporting Information

Additional supporting information can be found online in the Supporting Information section.

## Supporting information


**Supporting Information 1** Figure SI: (A) Rank abundance curve; (B) Shannon curve; (C) LDA scores of statistically different microbial communities in each group; (D) overlapping spectra of total ion current maps of QC samples in positive ion mode; (E) overlapping spectra of the total ion current maps of QC samples of negative ion mode; (F) multivariate analysis of significantly different metabolite expression between NC and DSS groups in the positive ion mode; (G) multivariate analysis of metabolite expression significantly different between NC and GEN groups in the positive ion mode; (H) hierarchical clustering heat maps of metabolites with significant differences within groups under positive ion mode; (I) hierarchical clustering heat map of metabolites with significant differences within groups under negative ion mode; (J) heat map of the Spearman correlation coefficient matrix of significantly different microflora and significantly different metabolites; and (K) a network graph showing a Spearman analysis of the link between significantly distinct microbiota and significantly distinct metabolites.

**Supporting Information 2** Research guideline checklist.

## Data Availability

The data that support the findings of this study are available from the corresponding author upon reasonable request.
