# Supplementary material for: Geniposide Alleviates Inflammatory Bowel Disease by Regulating Intestinal Flora and Arginine Metabolism and Inhibiting the NF‐κB Pathway Through Targeting Anxa5
Source: Mediators Inflamm. 2026 Jul 23;2026:6231832. doi: 10.1155/mi/6231832 (PMC13396694; doi:10.1155/mi/6231832)
Supplement: Supplementary file 1 — Supporting Information 1 Figure SI: (A) Rank abundance curve; (B) Shannon curve; (C) LDA scores of statistically different microbial communities in each group; (D) overlapping spectra of total ion current maps of QC samples in positive ion mode; (E) overlapping spectra of the total ion current maps of QC samples of negative ion mode; (F) multivariate analysis of significantly different metabolite expression between NC and DSS groups in the positive ion mode; (G) multivariate analysis of metabolite expression significantly different between NC and GEN groups in the positive ion mode; (H) hierarchical clustering heat maps of metabolites with significant differences within groups under positive ion mode; (I) hierarchical clustering heat map of metabolites with significant differences within groups under negative ion mode; (J) heat map of the Spearman correlation coefficient matrix of significantly different microflora and significantly different metabolites; and (K) a network graph showing a Spearman analysis of the link between significantly distinct microbiota and significantly distinct metabolites. [file MI-2026-6231832-s001.docx]

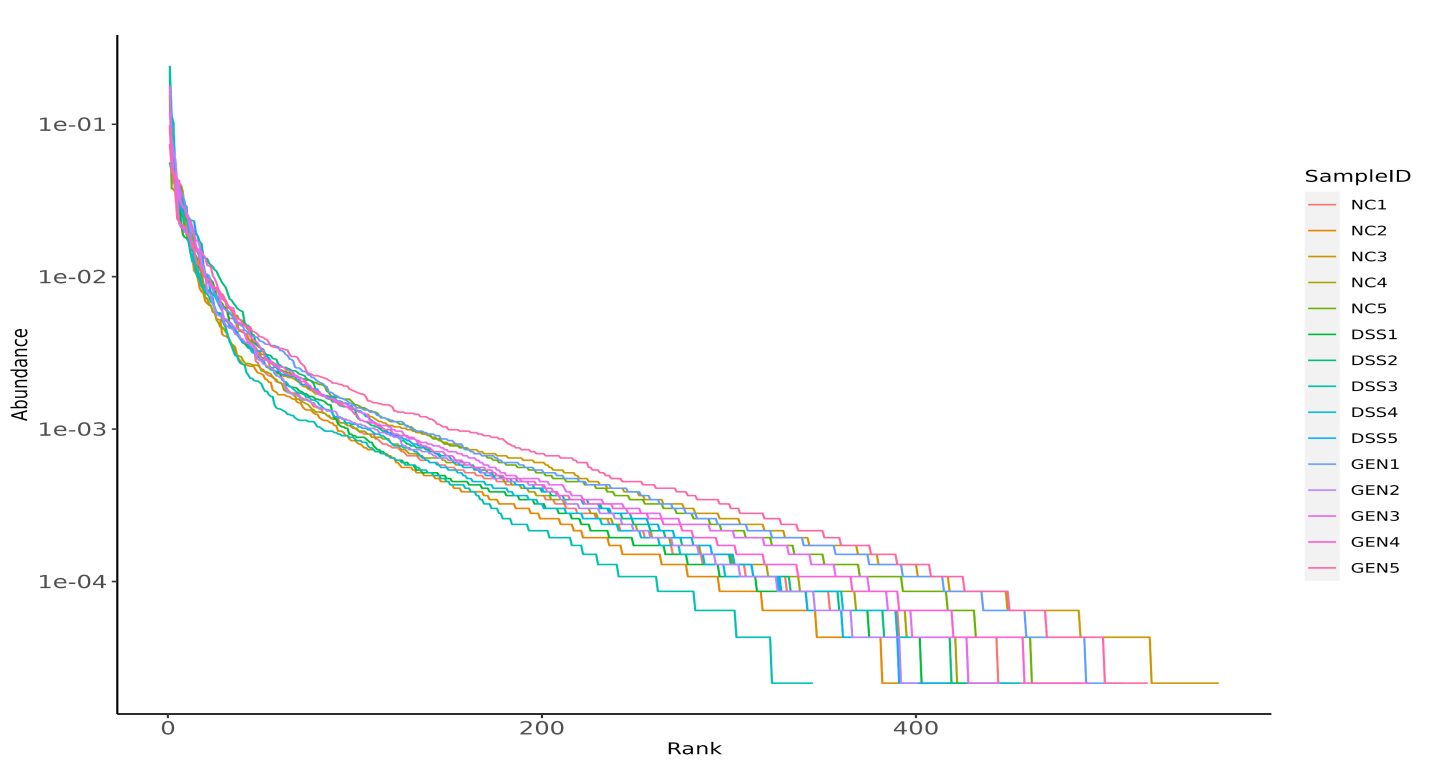


**S1A**

**S1B**


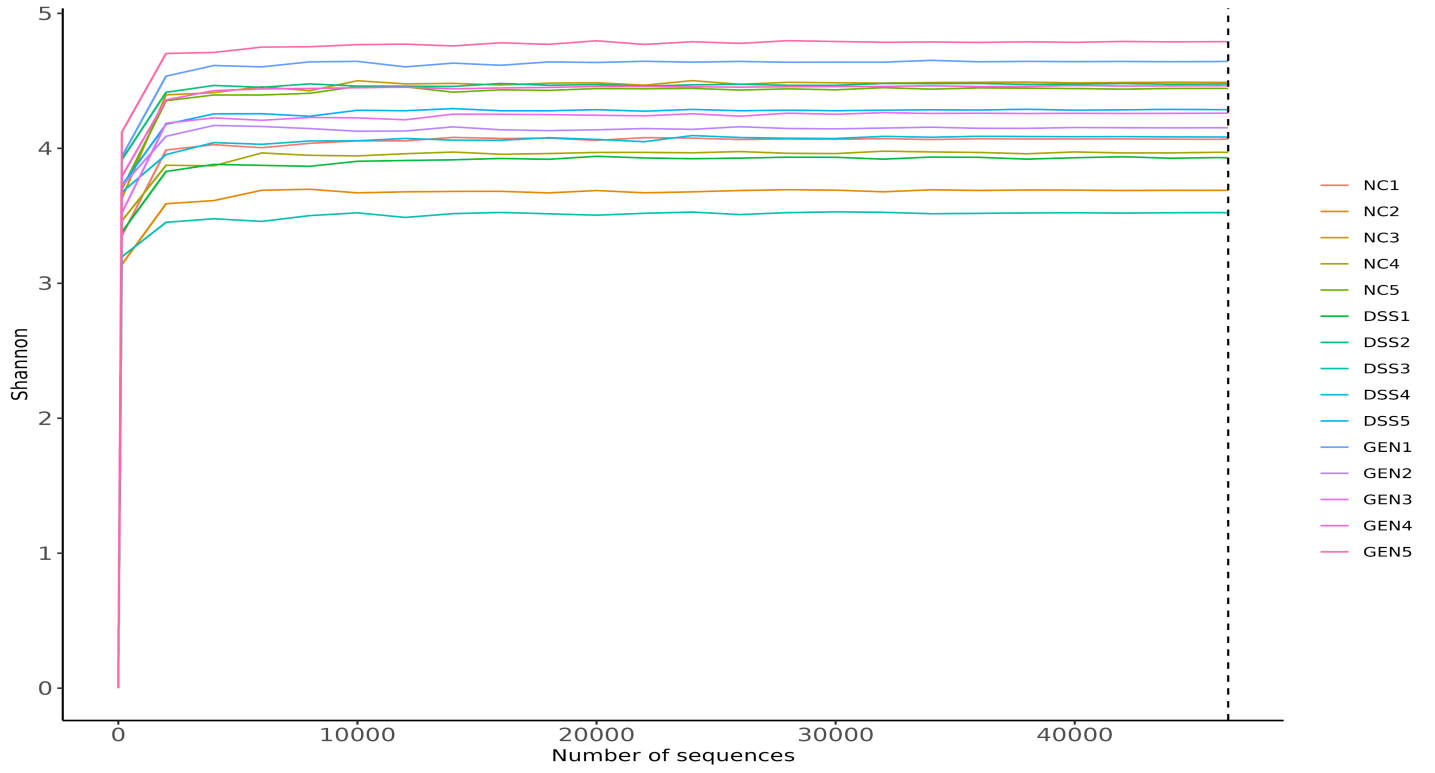


Figure SI. A, Rank abundance curve; B, Shannon curve

**S1C**


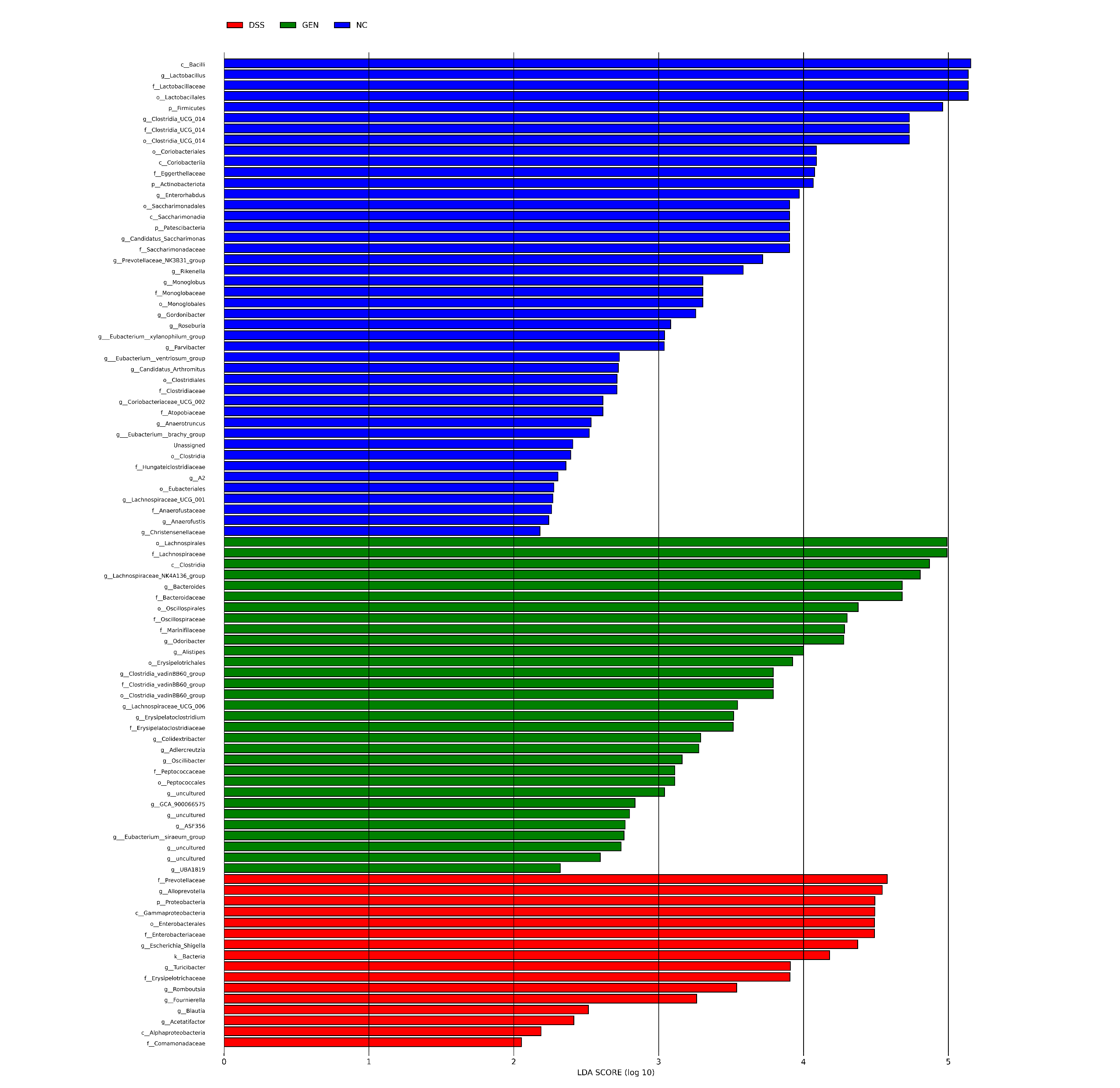


Figure S1. C, LDA scores of statistically different microbial communities in each group

**S1D**


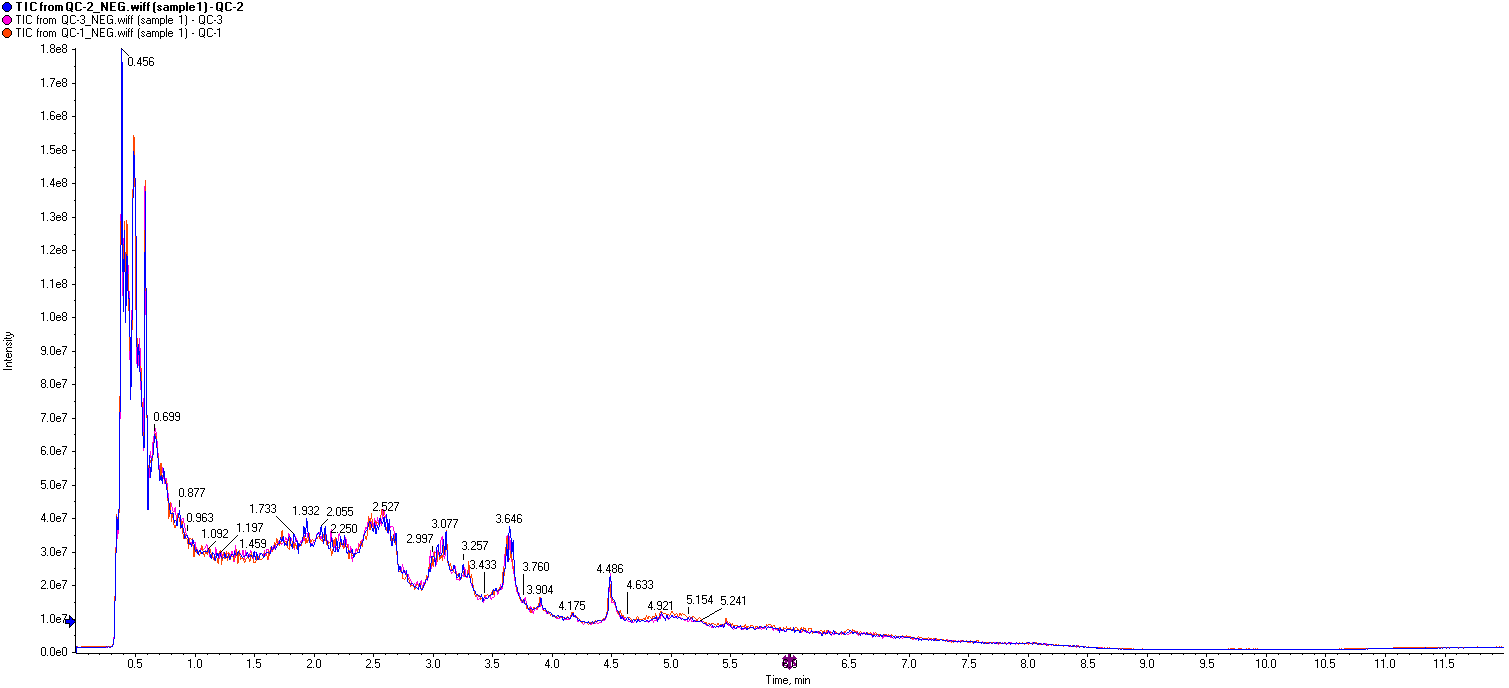

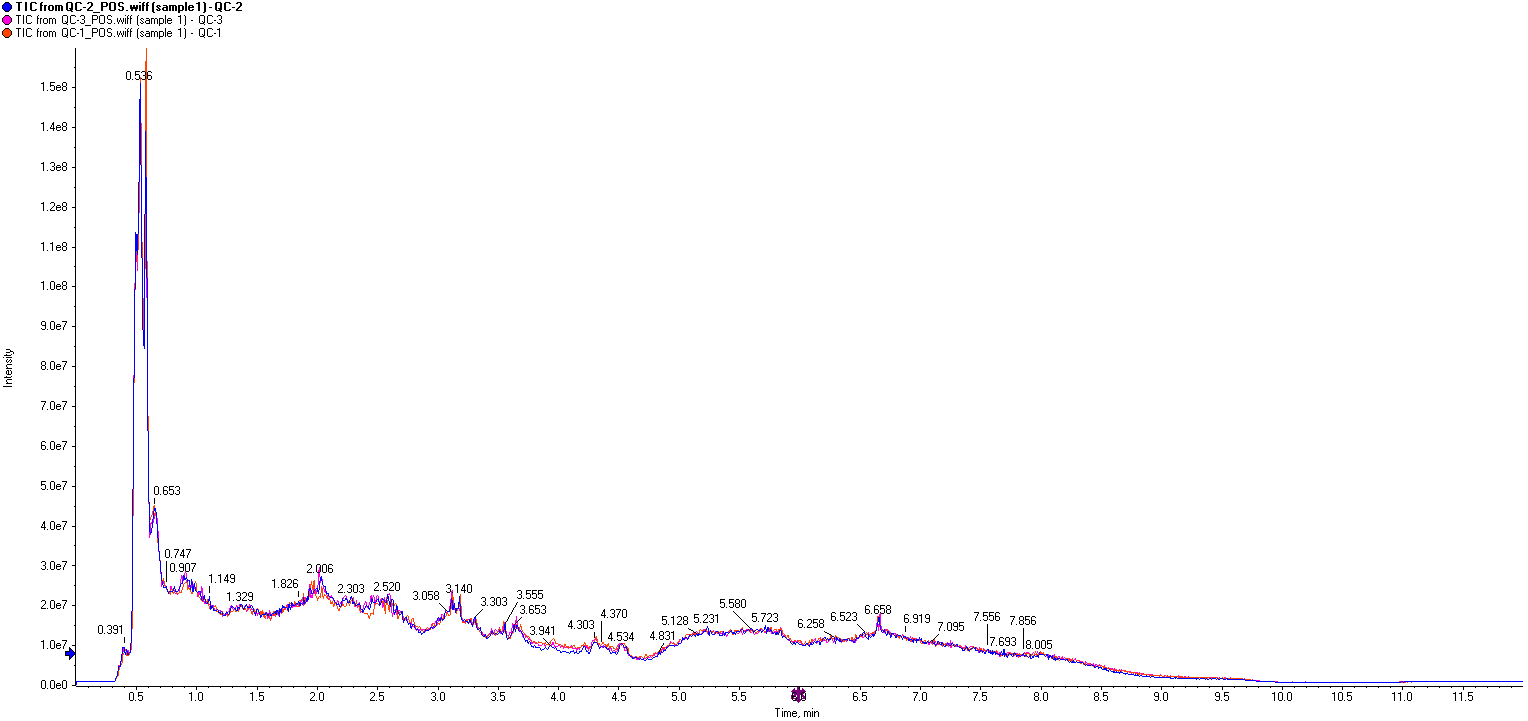


**S1E**

**S1F**


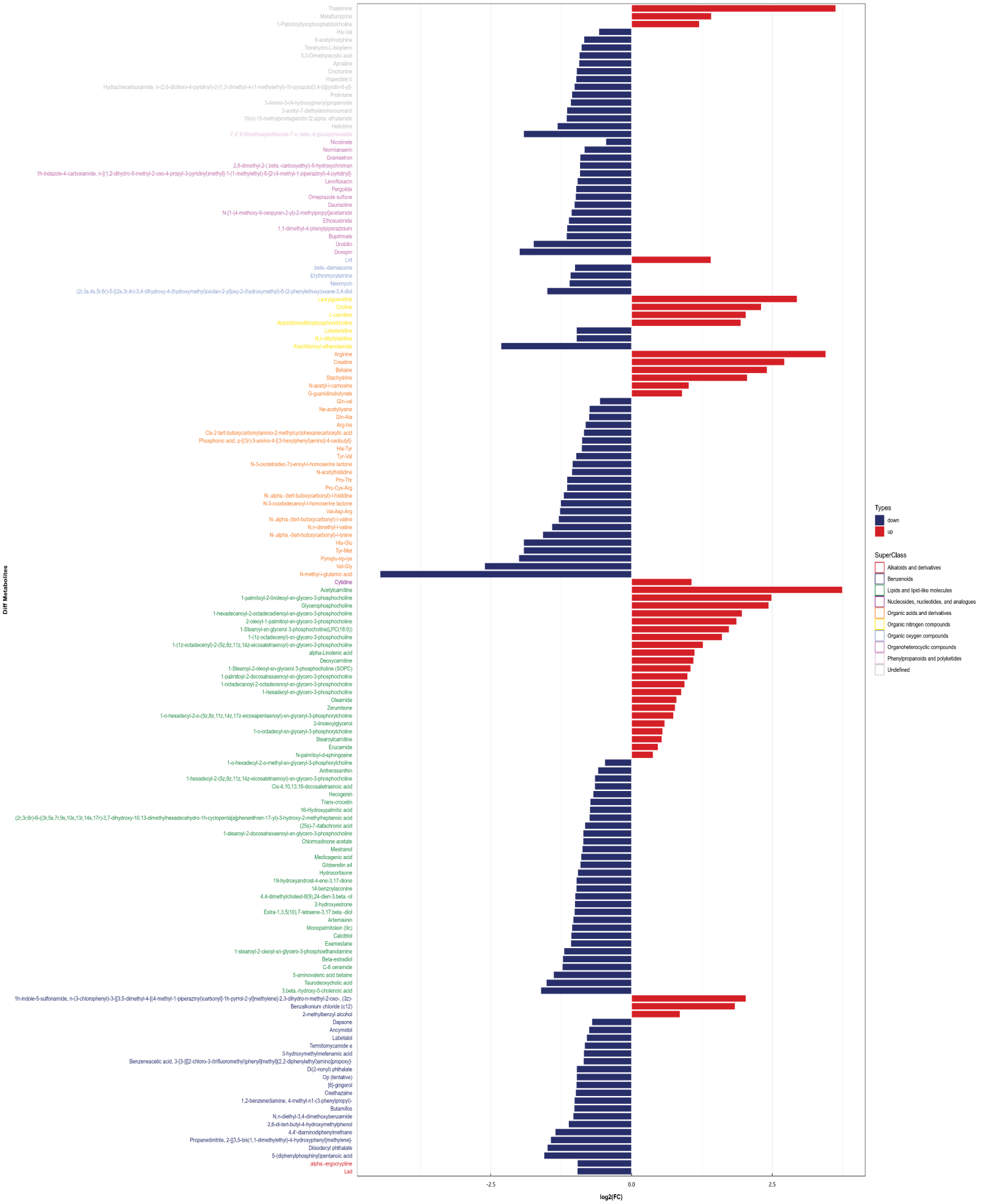


**S1G**


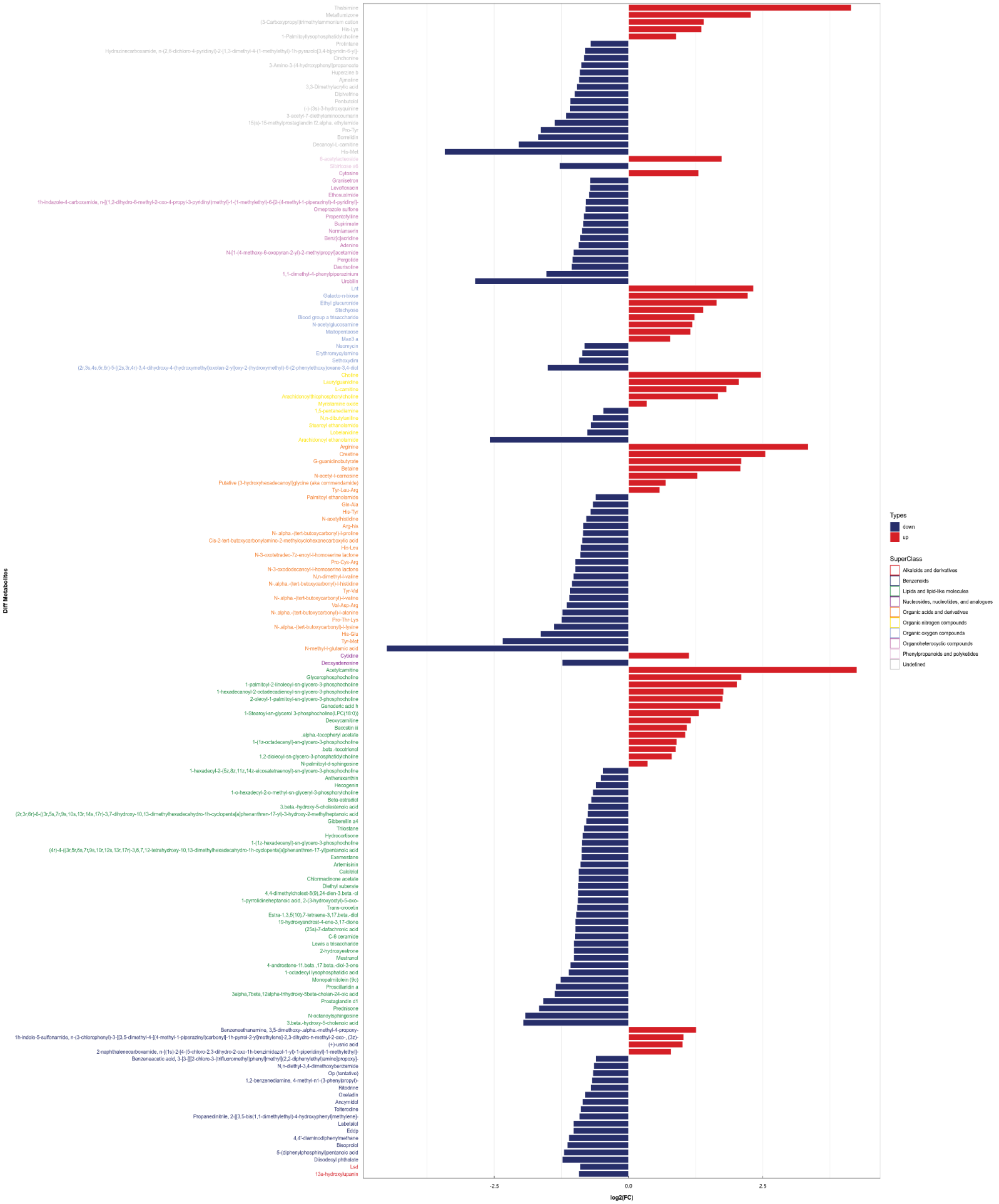


Figure S1. D, overlapping spectra of total ion current maps of QC samples in positive ion mode; E, overlapping spectra of the total ion current maps of QC samples of negative ion mode; F, multivariate analysis of significantly different metabolite expression between NC and DSS groups in the positive ion mode; G, multivariate analysis of metabolite expression significantly different between NC and GEN groups in the positive ion mode


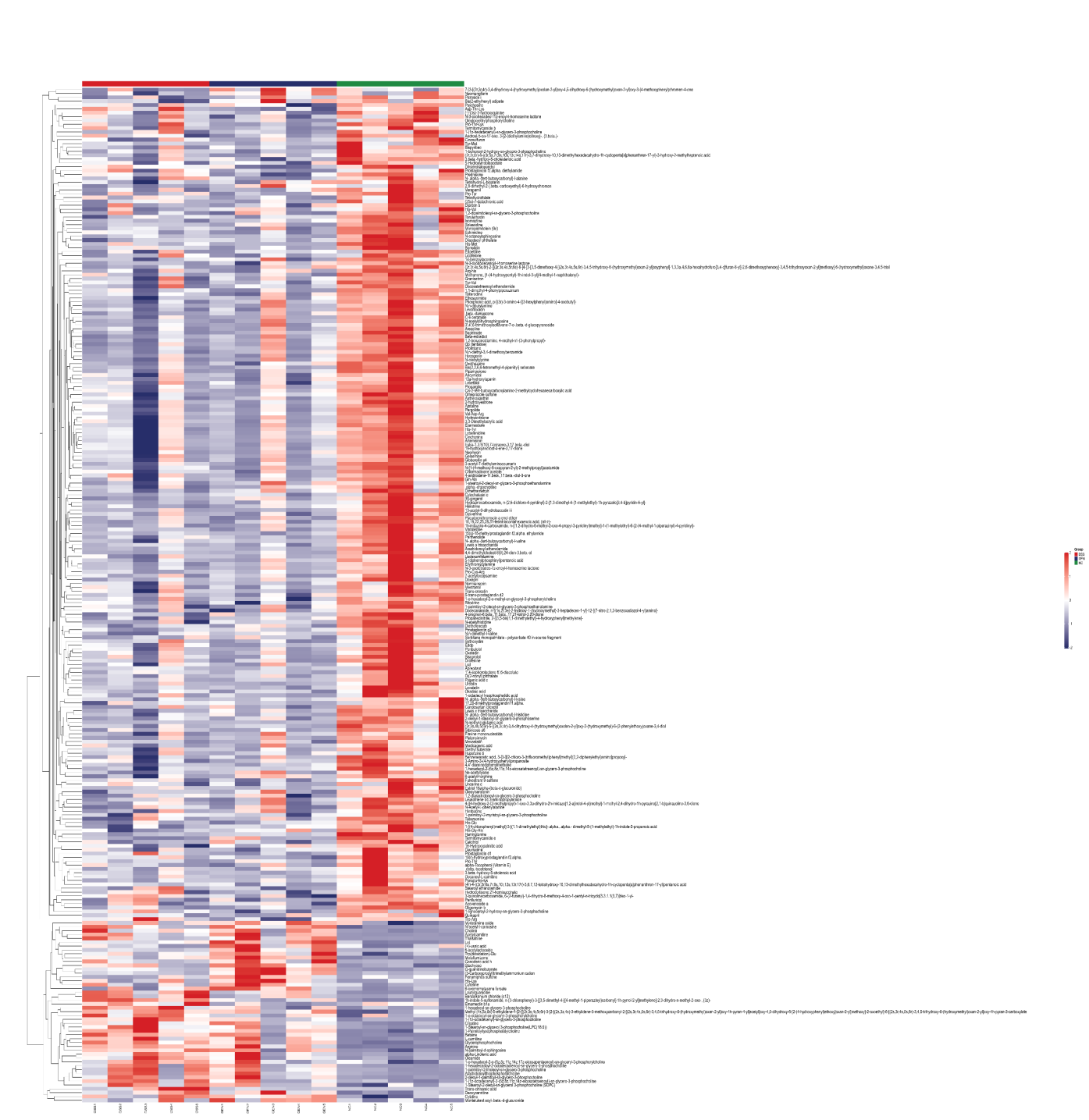


**S1H**


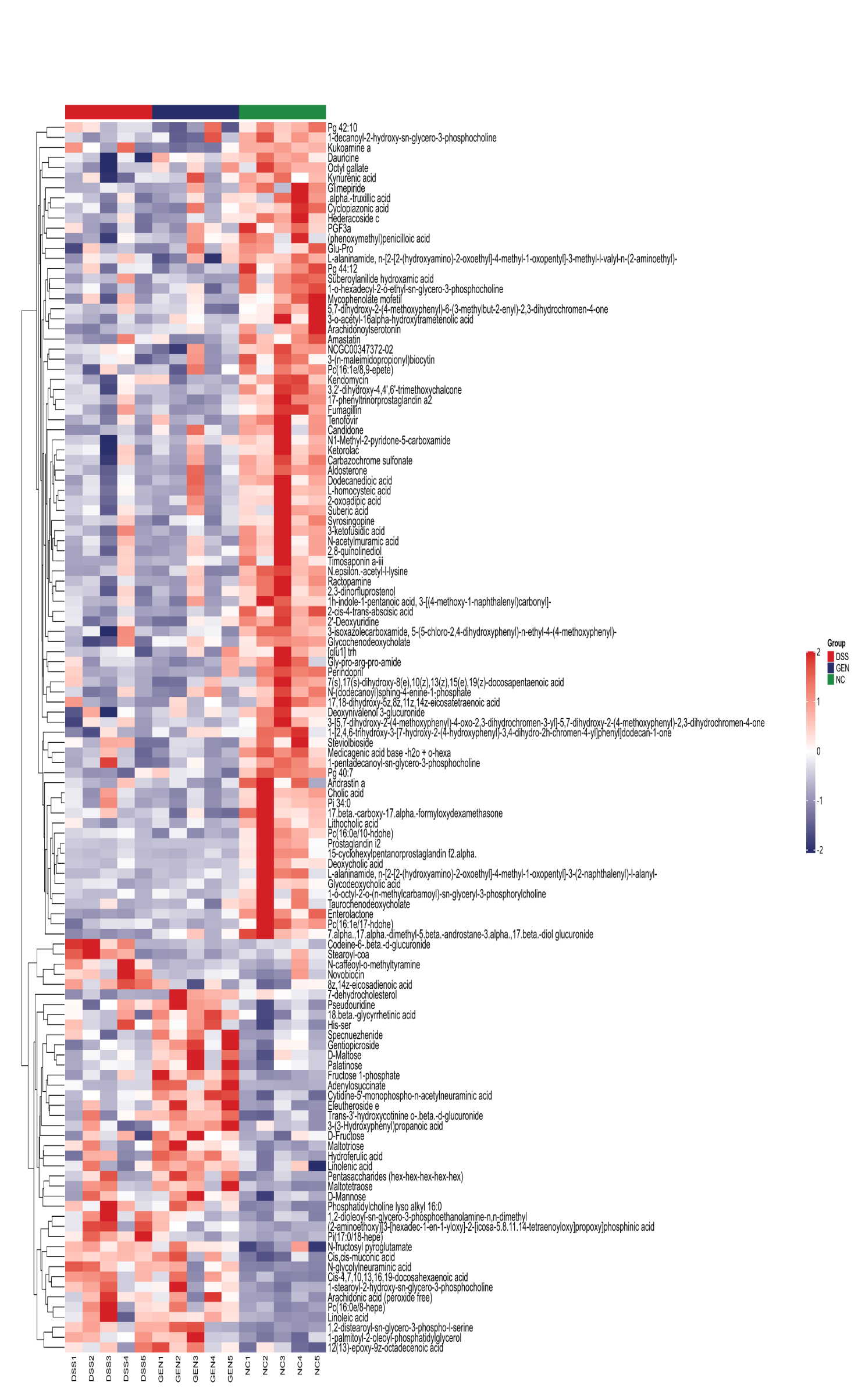


**S1I**

Figure S1. H, hierarchical clustering heat maps of metabolites with significant differences within groups under positive ion mode; I, hierarchical clustering heat map of metabolites with significant differences within groups under negative ion mode


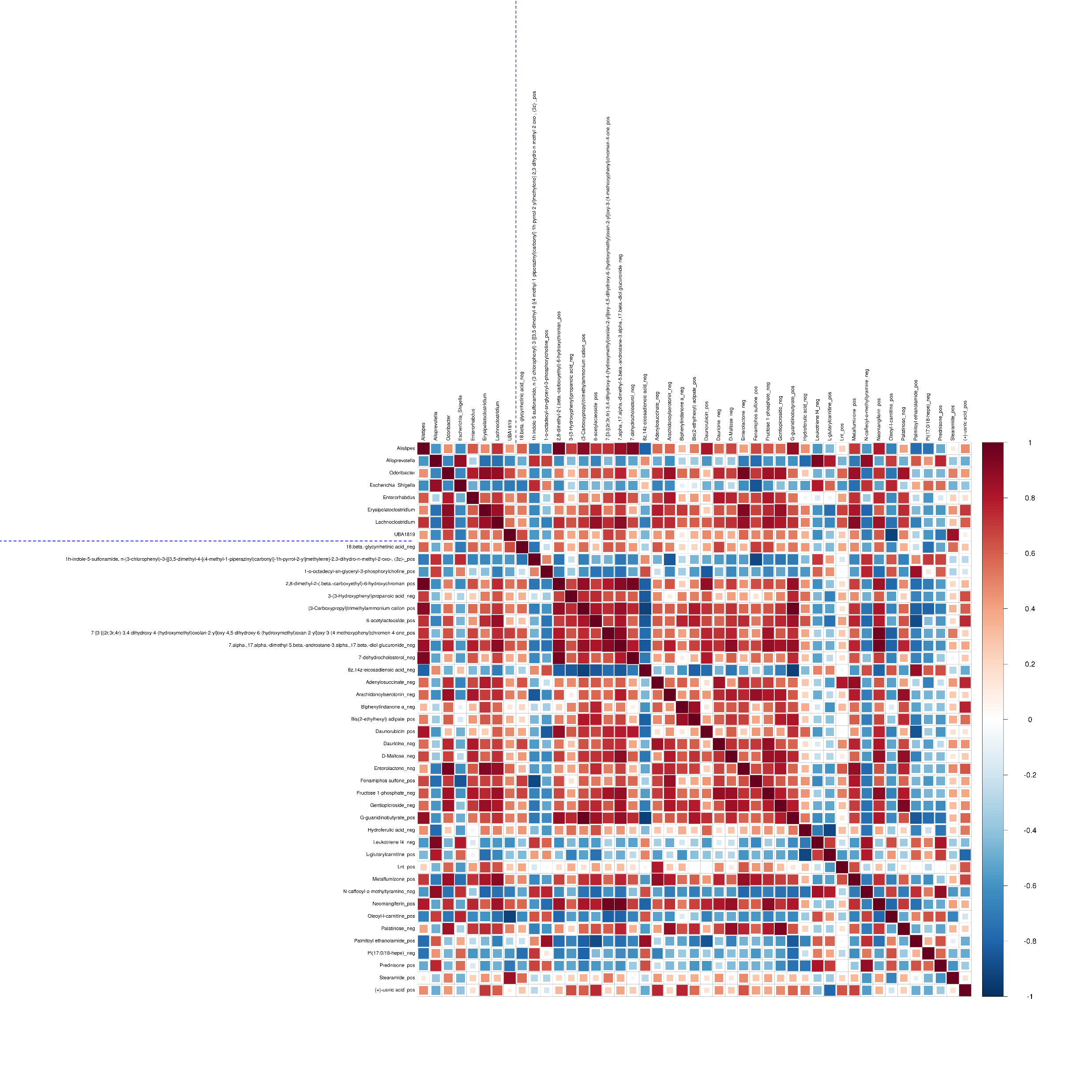


**S1J**

**S1K**


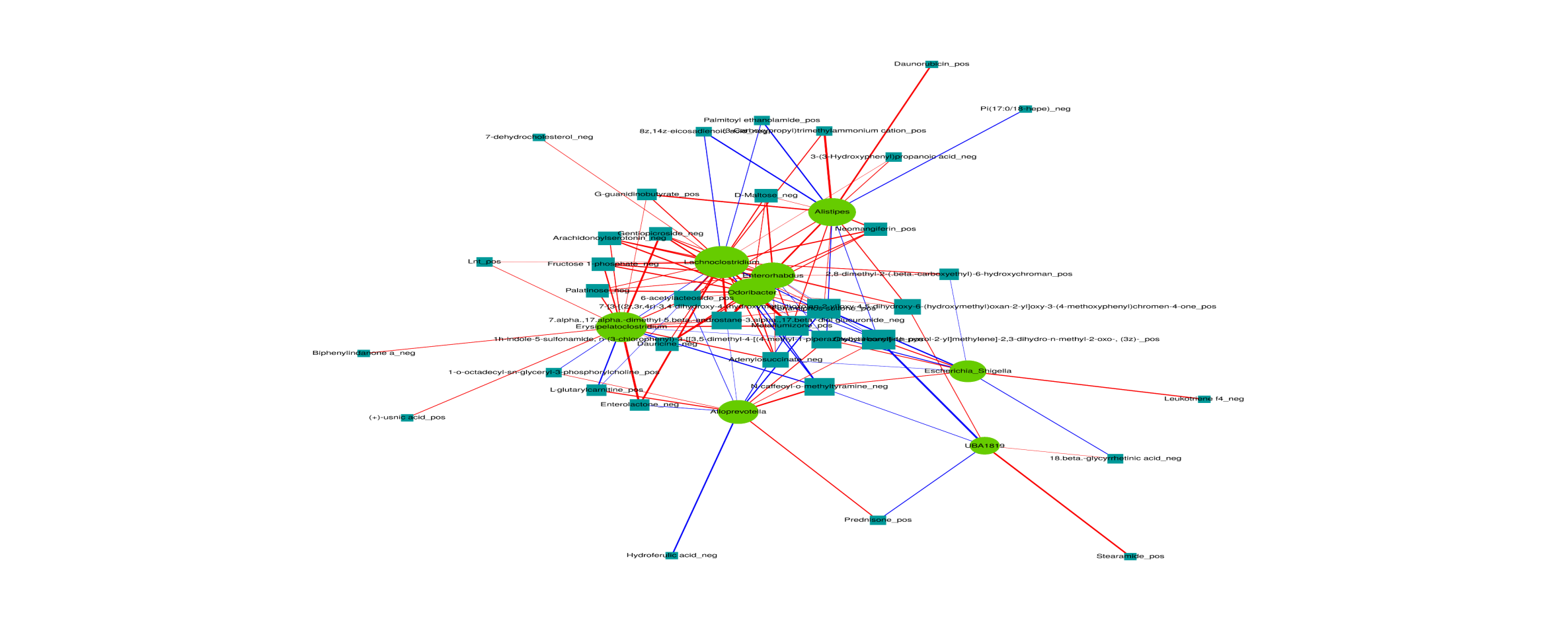


S1. J, heat map of the Spearman correlation coefficient matrix of significantly different microflora and significantly different metabolites; K, A network graph showing a Spearman analysis of the link between significantly distinct microbiota and significantly distinct metabolites
