# Supplementary material for: Geniposide Alleviates Inflammatory Bowel Disease by Regulating Intestinal Flora and Arginine Metabolism and Inhibiting the NF‐κB Pathway Through Targeting Anxa5
Source: Mediators Inflamm. 2026 Jul 23;2026:6231832. doi: 10.1155/mi/6231832 (PMC13396694; doi:10.1155/mi/6231832)
Supplement: Supplementary file 2 — Supporting Information 2 Research guideline checklist. [file MI-2026-6231832-s002.pdf]

S1A

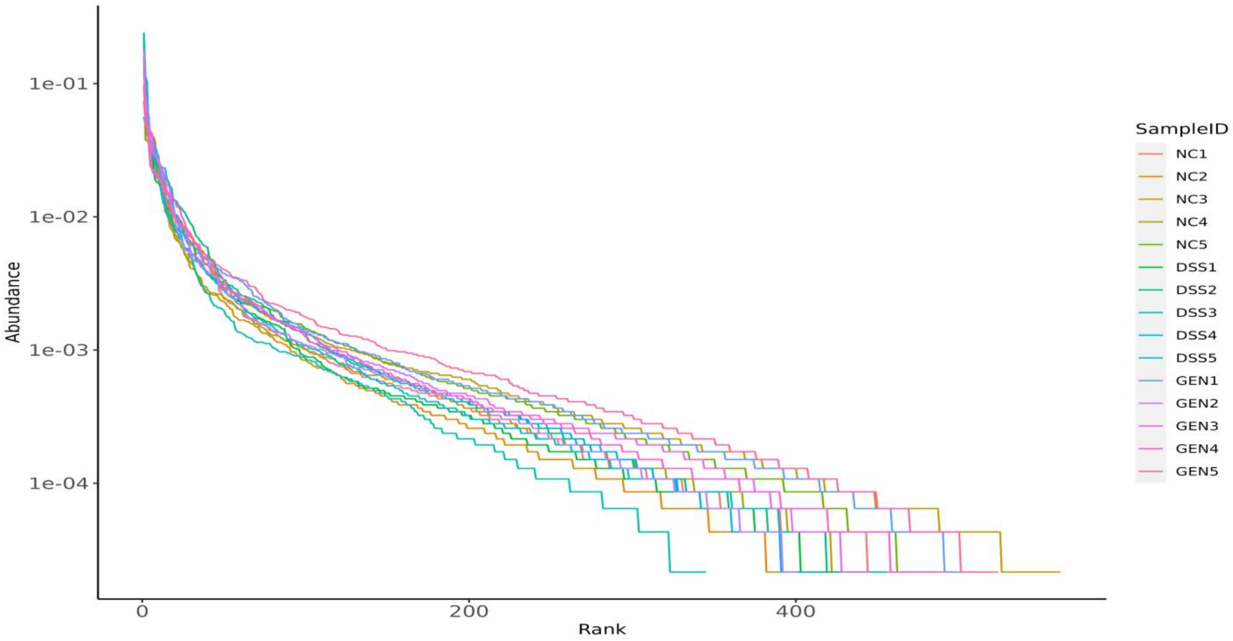

S1B

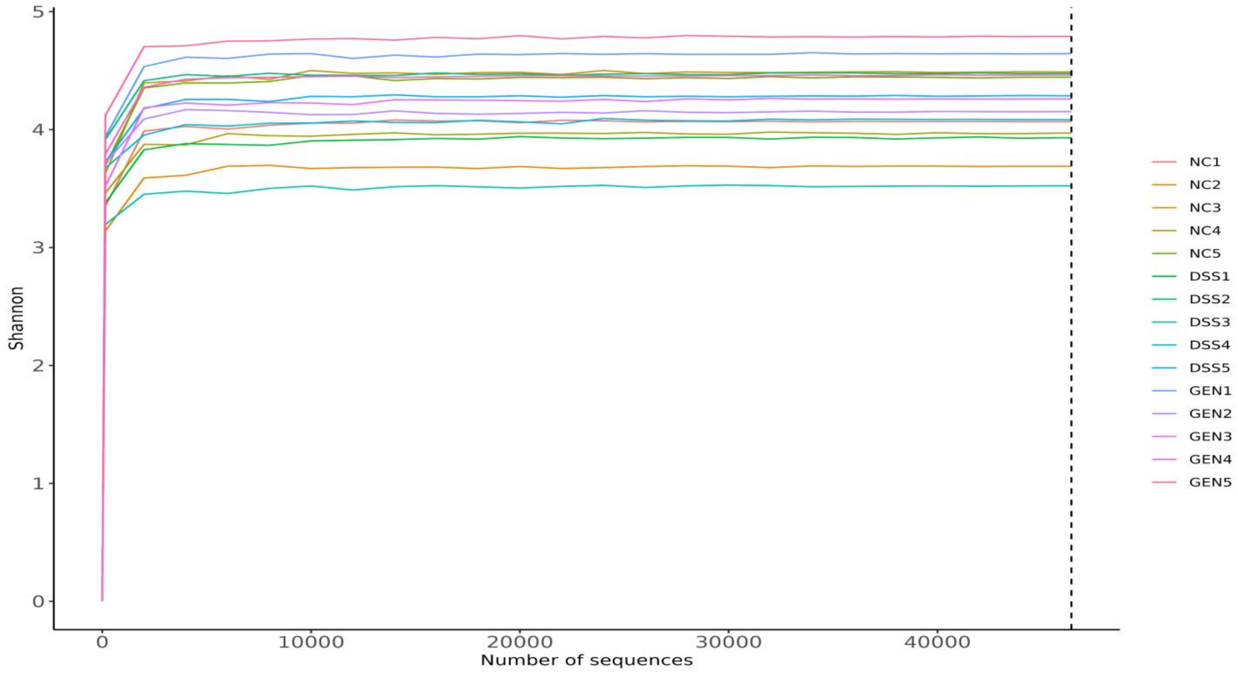

Figure SI. A, Rank abundance curve; B, Shannon curve

S1C

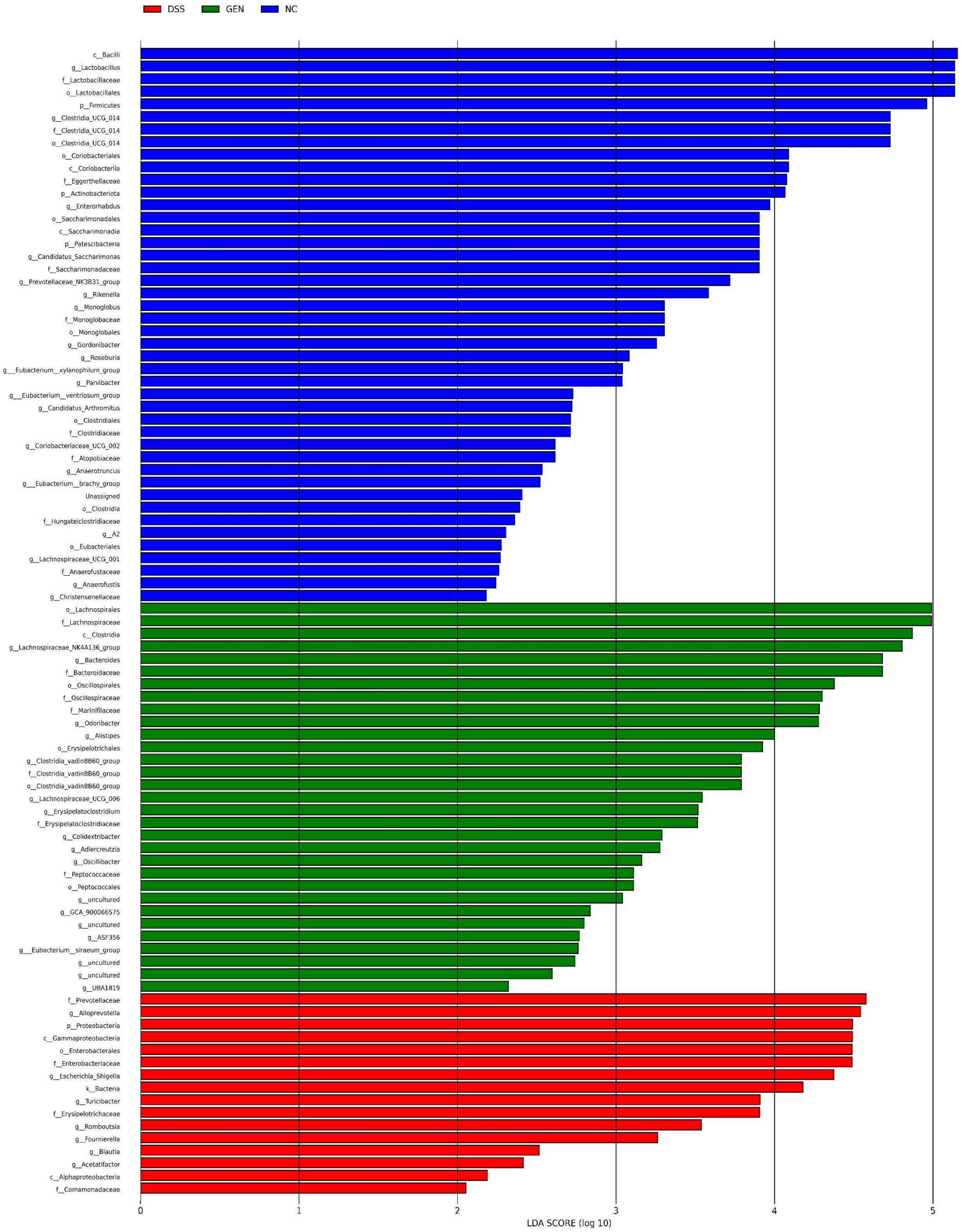

Figure S1. C, LDA scores of statistically different microbial communities in each group

S1D

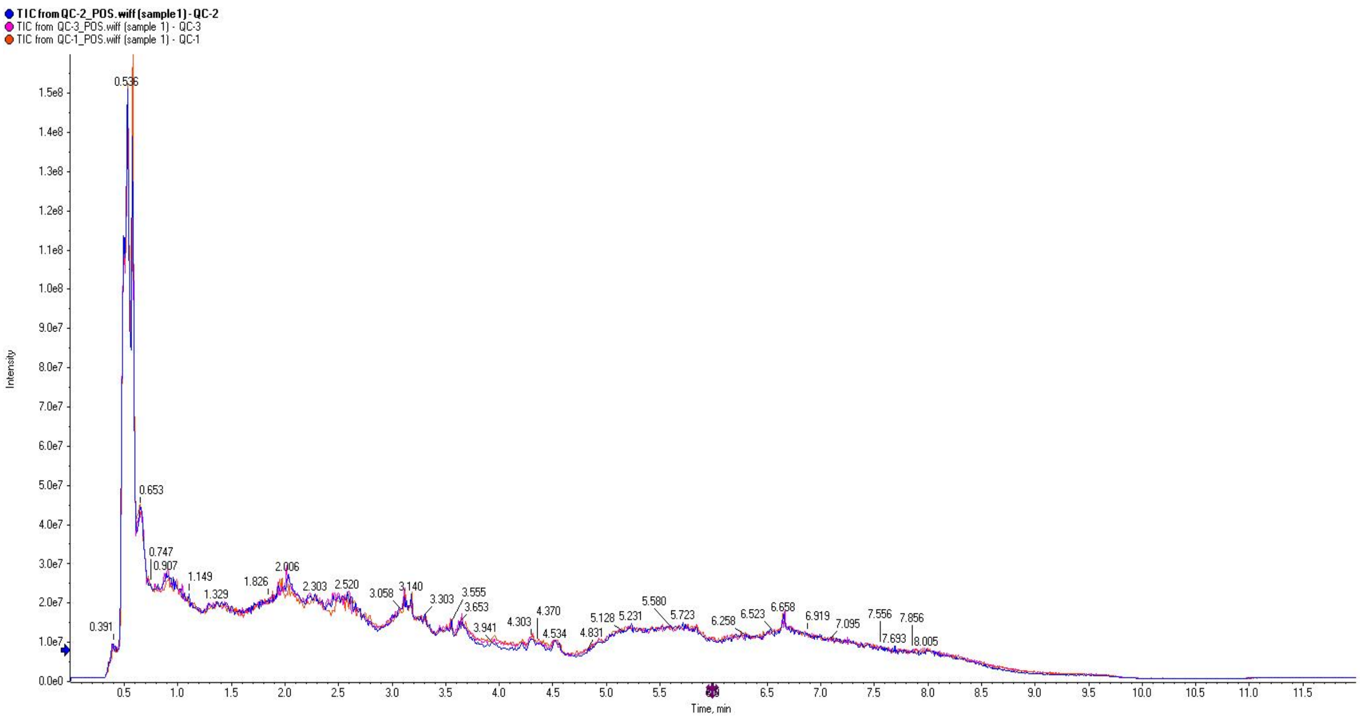
TIC from QC-2_POS.wiff (sample1) - QC-2
TIC from QC-3_POS.wiff (sample 1) - QC-3
TIC from QC-1_POS.wiff (sample 1) - QC-1
Intensity
Time, min

S1E

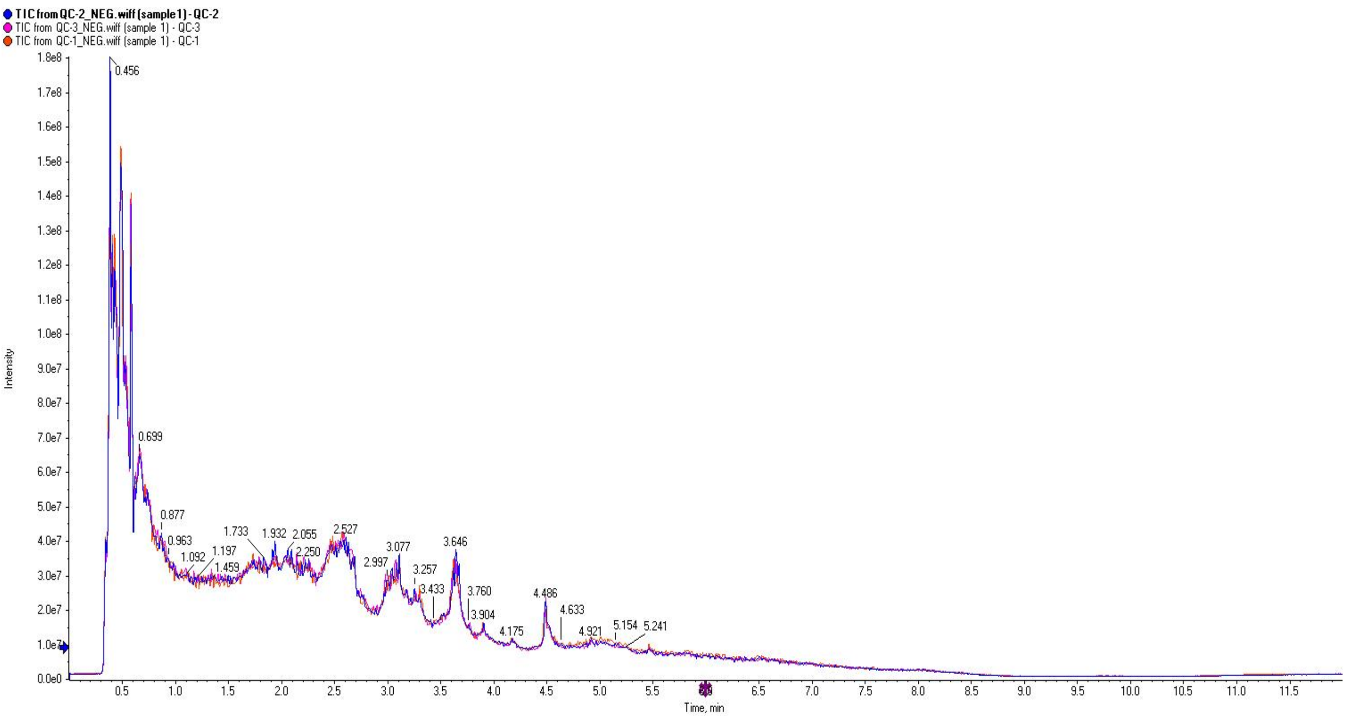
TIC from QC-2_NEG.wiff (sample1) - QC-2
TIC from QC-3_NEG.wiff (sample 1) - QC-3
TIC from QC-1_NEG.wiff (sample 1) - QC-1
Intensity
Time, min

S1F

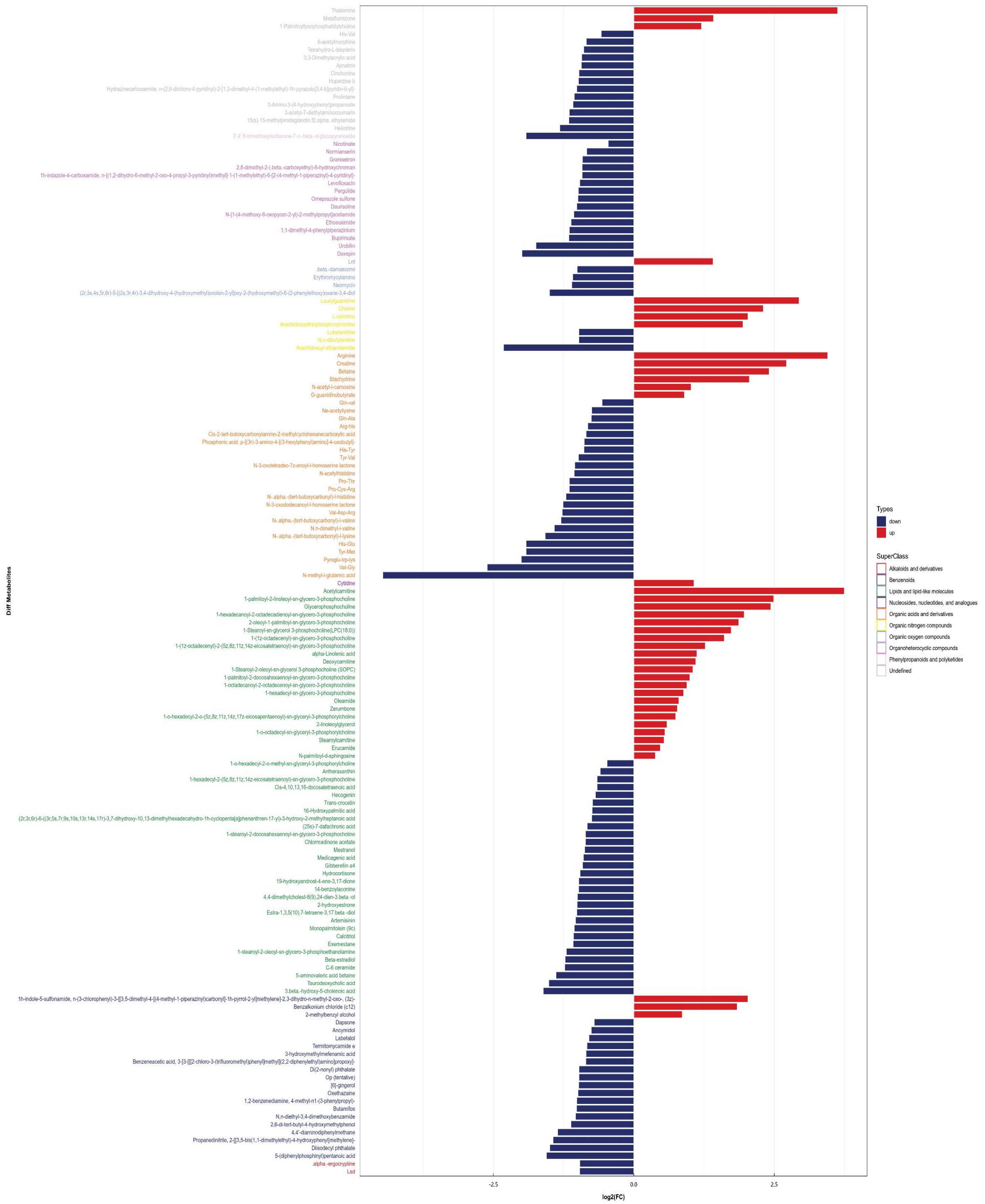
Thalsimine
Metaflumizone
1-Palmitoyllysophosphatidylcholine
His-Val
6-acetylmorphine
Tetrahydro-L-biopterin
3,3-Dimethylacrylic acid
Ajmaline
Cinchonine
Huperzine b
Hydrazinecarboxamide, n-(2,6-dichloro-4-pyridinyl)-2-[1,3-dimethyl-4-(1-methylethyl)-1h-pyrazolo[3,4-b]pyridin-6-yl]-
Prolintane
3-Amino-3-(4-hydroxyphenyl)propanoate
3-acetyl-7-diethylaminocoumarin
15(s)-15-methylprostaglandin f2.alpha. ethylamide
Heliotrine
3',4',6-trimethoxyisoflavone-7-o-.beta.-d-glucopyranoside
Nicotinate
Normianserin
Granisetron
2,8-dimethyl-2-(.beta.-carboxyethyl)-6-hydroxychroman
1h-indazole-4-carboxamide, n-[(1,2-dihydro-6-methyl-2-oxo-4-propyl-3-pyridinyl)methyl]-1-(1-methylethyl)-6-[2-(4-methyl-1-piperazinyl)-4-pyridinyl]-
Levofloxacin
Pergolide
Omeprazole sulfone
Daurisoline
N-[1-(4-methoxy-6-oxopyran-2-yl)-2-methylpropyl]acetamide
Ethosuximide
1,1-dimethyl-4-phenylpiperazinium
Bupirimate
Urobilin
Doxepin
Lnt
.beta.-damascone
Erythromycylamine
Neomycin
(2r,3s,4s,5r,6r)-5-[(2s,3r,4r)-3,4-dihydroxy-4-(hydroxymethyl)oxolan-2-yl]oxy-2-(hydroxymethyl)-6-(2-phenylethoxy)oxane-3,4-diol
Laurylguanidine
Choline
L-carnitine
Arachidonoylthiophosphorylcholine
Lobelanidine
N,n-dibutylaniline
Arachidonoyl ethanolamide
Arginine
Creatine
Betaine
Stachydrine
N-acetyl-l-carnosine
G-guanidinobutyrate
Gln-val
Ne-acetyllysine
Gln-Ala
Arg-his
Cis-2-tert-butoxycarbonylamino-2-methylcyclohexanecarboxylic acid
Phosphonic acid, p-[(3r)-3-amino-4-[(3-hexylphenyl)amino]-4-oxobutyl]-
His-Tyr
Tyr-Val
N-3-oxotetradec-7z-enoyl-l-homoserine lactone
N-acetylhistidine
Pro-Thr
Pro-Cys-Arg
N-.alpha.-(tert-butoxycarbonyl)-l-histidine
N-3-oxododecanoyl-l-homoserine lactone
Val-Asp-Arg
N-.alpha.-(tert-butoxycarbonyl)-l-valine
N,n-dimethyl-l-valine
N-.alpha.-(tert-butoxycarbonyl)-l-lysine
His-Glu
Tyr-Met
Pyroglu-trp-lys
Val-Gly
N-methyl-l-glutamic acid
Cytidine
Acetylcarnitine
1-palmitoyl-2-linoleoyl-sn-glycero-3-phosphocholine
Glycerophosphocholine
1-hexadecanoyl-2-octadecadienoyl-sn-glycero-3-phosphocholine
2-oleoyl-1-palmitoyl-sn-glycero-3-phosphocholine
1-Stearoyl-sn-glycerol 3-phosphocholine(LPC(18:0))
1-(1z-octadecenyl)-sn-glycero-3-phosphocholine
1-(1z-octadecenyl)-2-(5z,8z,11z,14z-eicosatetraenoyl)-sn-glycero-3-phosphocholine
alpha-Linolenic acid
Deoxycarnitine
1-Stearoyl-2-oleoyl-sn-glycerol 3-phosphocholine (SOPC)
1-palmitoyl-2-docosahexaenoyl-sn-glycero-3-phosphocholine
1-octadecanoyl-2-octadecenoyl-sn-glycero-3-phosphocholine
1-hexadecyl-sn-glycero-3-phosphocholine
Oleamide
Zerumbone
1-o-hexadecyl-2-o-(5z,8z,11z,14z,17z-eicosapentaenoyl)-sn-glyceryl-3-phosphorylcholine
2-linoleoylglycerol
1-o-octadecyl-sn-glyceryl-3-phosphorylcholine
Stearoylcarnitine
Erucamide
N-palmitoyl-d-sphingosine
1-o-hexadecyl-2-o-methyl-sn-glyceryl-3-phosphorylcholine
Antheraxanthin
1-hexadecyl-2-(5z,8z,11z,14z-eicosatetraenoyl)-sn-glycero-3-phosphocholine
Cis-4,10,13,16-docosatetraenoic acid
Hecogenin
Trans-crocetin
16-Hydroxypalmitic acid
(2r,3r,6r)-6-((3r,5s,7r,9s,10s,13r,14s,17r)-3,7-dihydroxy-10,13-dimethylhexadecahydro-1h-cyclopenta[a]phenanthren-17-yl)-3-hydroxy-2-methylheptanoic acid
(25s)-7-dafachronic acid
1-stearoyl-2-docosahexaenoyl-sn-glycero-3-phosphocholine
Chlormadinone acetate
Mestranol
Medicagenic acid
Gibberellin a4
Hydrocortisone
19-hydroxyandrost-4-ene-3,17-dione
14-benzoylaconine
4,4-dimethylcholest-8(9),24-dien-3.beta.-ol
2-hydroxyestrone
Estra-1,3,5(10),7-tetraene-3,17.beta.-diol
Artemisinin
Monopalmitolein (9c)
Calcitriol
Exemestane
1-stearoyl-2-oleoyl-sn-glycero-3-phosphoethanolamine
Beta-estradiol
C-6 ceramide
5-aminovaleric acid betaine
Taurodeoxycholic acid
3.beta.-hydroxy-5-cholenoic acid
1h-indole-5-sulfonamide, n-(3-chlorophenyl)-3-[[3,5-dimethyl-4-[(4-methyl-1-piperazinyl)carbonyl]-1h-pyrrol-2-yl]methylene]-2,3-dihydro-n-methyl-2-oxo-, (3z)-
Benzalkonium chloride (c12)
2-methylbenzyl alcohol
Dapsone
Ancymidol
Labetalol
Termitomycamide e
3-hydroxymethylmefenamic acid
Benzeneacetic acid, 3-[3-[[[2-chloro-3-(trifluoromethyl)phenyl]methyl](2,2-diphenylethyl)amino]propoxy]-
Di(2-nonyl) phthalate
Op (tentative)
[6]-gingerol
Oxethazaine
1,2-benzenediamine, 4-methyl-n1-(3-phenylpropyl)-
Butamifos
N,n-diethyl-3,4-dimethoxybenzamide
2,6-di-tert-butyl-4-hydroxymethylphenol
4,4'-diaminodiphenylmethane
Propanedinitrile, 2-[[3,5-bis(1,1-dimethylethyl)-4-hydroxyphenyl]methylene]-
Diisodecyl phthalate
5-(diphenylphosphinyl)pentanoic acid
.alpha.-ergocryptine
Lsd
Diff Metabolites
-2.5
0.0
2.5
log2(FC)
Types
down
up
SuperClass
Alkaloids and derivatives
Benzenoids
Lipids and lipid-like molecules
Nucleosides, nucleotides, and analogues
Organic acids and derivatives
Organic nitrogen compounds
Organic oxygen compounds
Organoheterocyclic compounds
Phenylpropanoids and polyketides
Undefined

S1G

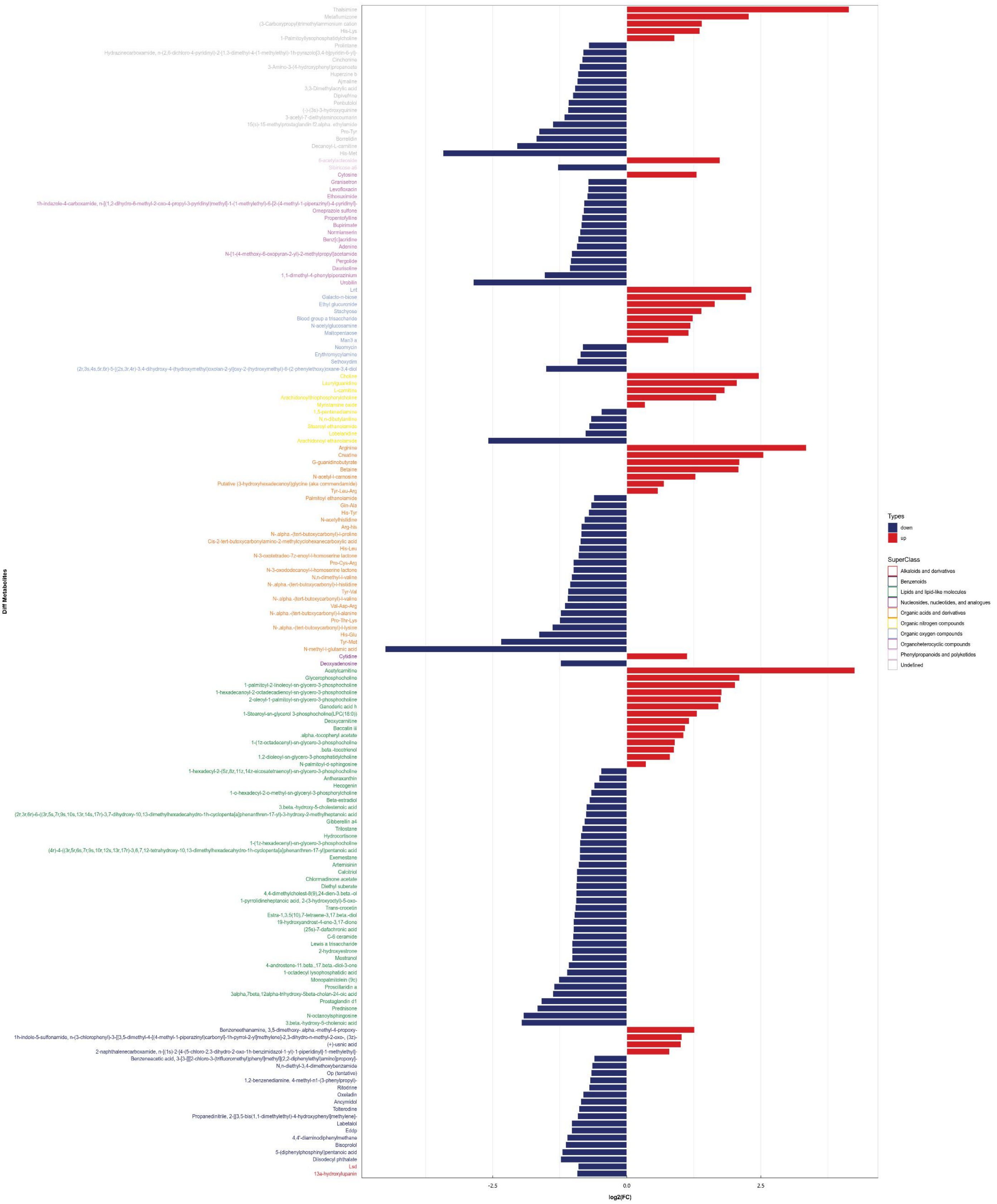
Diff Metabolites
log2(FC)
-2.5
0.0
2.5
Types
down
up
SuperClass
Alkaloids and derivatives
Benzenoids
Lipids and lipid-like molecules
Nucleosides, nucleotides, and analogues
Organic acids and derivatives
Organic nitrogen compounds
Organic oxygen compounds
Organoheterocyclic compounds
Phenylpropanoids and polyketides
Undefined

Figure S1. D, overlapping spectra of total ion current maps of QC samples in positive ion mode; E, overlapping spectra of the total ion current maps of QC samples of negative ion mode; F, multivariate analysis of significantly different metabolite expression between NC and DSS groups in the positive ion mode; G, multivariate analysis of metabolite expression significantly different between NC and GEN groups in the positive ion mode

S1H

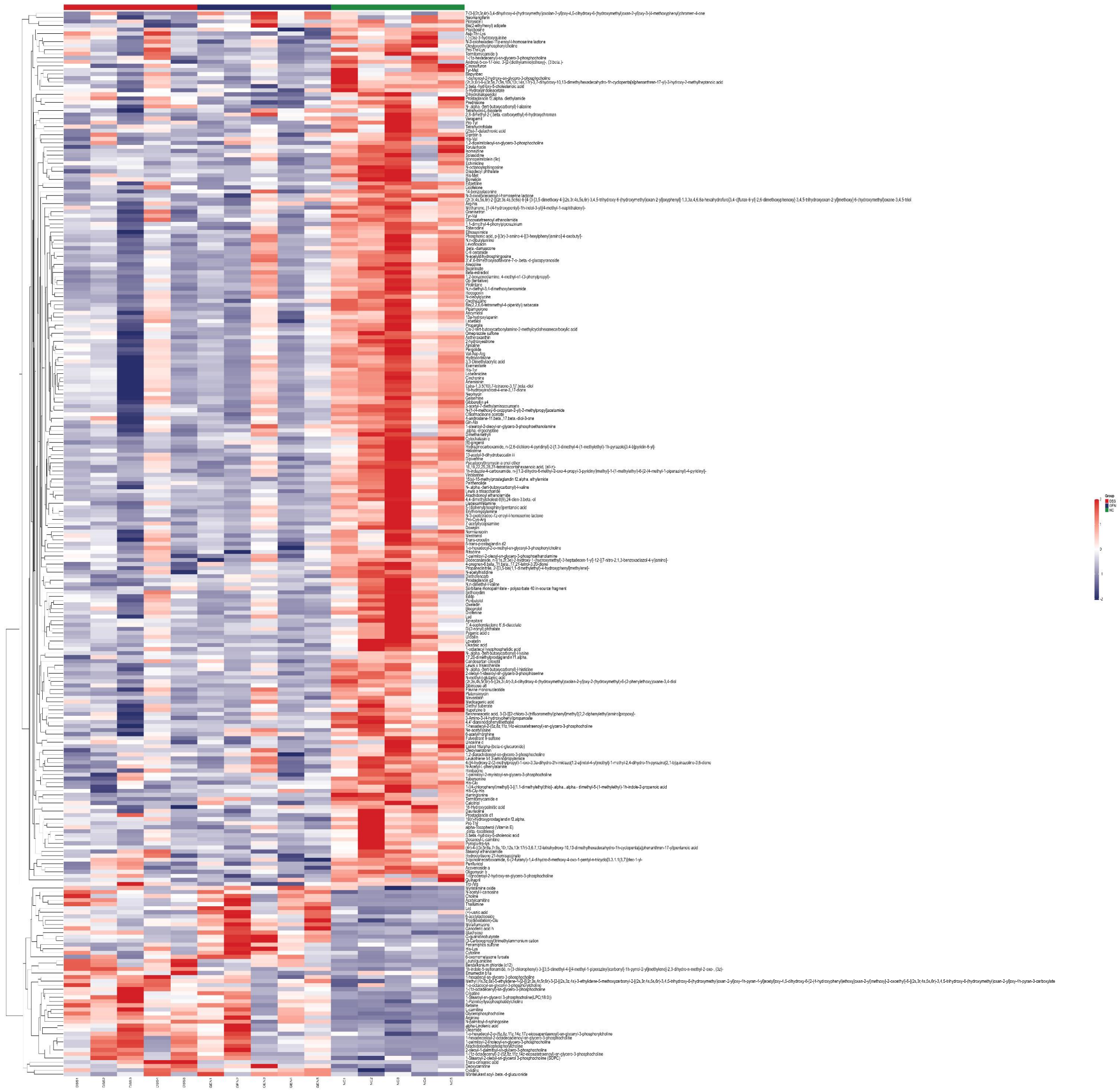

S1I

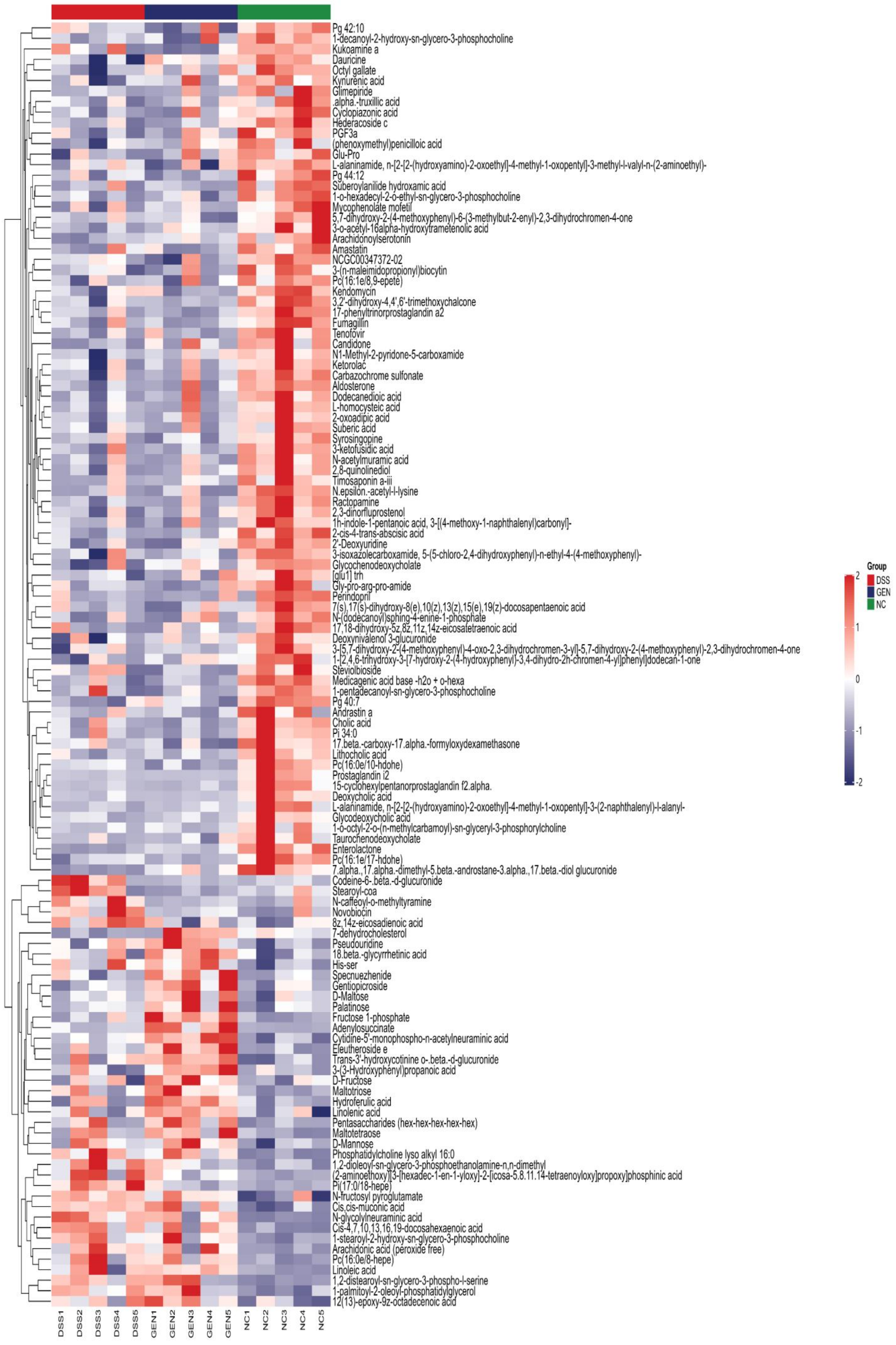
Pg 42:10
1-decanoyl-2-hydroxy-sn-glycero-3-phosphocholine
Kukoamine a
Dauricine
Octyl gallate
Kynurenic acid
Glimepiride
.alpha.-truxillic acid
Cyclopiazonic acid
Hederacoside c
PGF3a
(phenoxymethyl)penicilloic acid
Glu-Pro
L-alaninamide, n-[2-[2-(hydroxyamino)-2-oxoethyl]-4-methyl-1-oxopentyl]-3-methyl-l-valyl-n-(2-aminoethyl)-
Pg 44:12
Suberoylanilide hydroxamic acid
1-o-hexadecyl-2-o-ethyl-sn-glycero-3-phosphocholine
Mycophenolate mofetil
5,7-dihydroxy-2-(4-methoxyphenyl)-6-(3-methylbut-2-enyl)-2,3-dihydrochromen-4-one
3-o-acetyl-16alpha-hydroxytrametenolic acid
Arachidonoylserotonin
Amastatin
NCGC00347372-02
3-(n-maleimidopropionyl)biocytin
Pc(16:1e/8,9-epete)
Kendomycin
3,2'-dihydroxy-4,4',6'-trimethoxychalcone
17-phenyltrinorprostaglandin a2
Fumagillin
Tenofovir
Candidone
N1-Methyl-2-pyridone-5-carboxamide
Ketorolac
Carbazochrome sulfonate
Aldosterone
Dodecanedioic acid
L-homocysteic acid
2-oxoadipic acid
Suberic acid
Syrosingopine
3-ketofusidic acid
N-acetylmuramic acid
2,8-quinolinediol
Timosaponin a-iii
N.epsilon.-acetyl-l-lysine
Ractopamine
2,3-dinorfluprostenol
1h-indole-1-pentanoic acid, 3-[(4-methoxy-1-naphthalenyl)carbonyl]-
2-cis-4-trans-abscisic acid
2'-Deoxyuridine
3-isoxazolecarboxamide, 5-(5-chloro-2,4-dihydroxyphenyl)-n-ethyl-4-(4-methoxyphenyl)-
Glycochenodeoxycholate
[glu1] trh
Gly-pro-arg-pro-amide
Perindopril
7(s),17(s)-dihydroxy-8(e),10(z),13(z),15(e),19(z)-docosapentaenoic acid
N-(dodecanoyl)sphing-4-enine-1-phosphate
17,18-dihydroxy-5z,8z,11z,14z-eicosatetraenoic acid
Deoxynivalenol 3-glucuronide
3-[5,7-dihydroxy-2-(4-methoxyphenyl)-4-oxo-2,3-dihydrochromen-3-yl]-5,7-dihydroxy-2-(4-methoxyphenyl)-2,3-dihydrochromen-4-one
1-[2,4,6-trihydroxy-3-[7-hydroxy-2-(4-hydroxyphenyl)-3,4-dihydro-2h-chromen-4-yl]phenyl]dodecan-1-one
Steviolbioside
Medicagenic acid base -h2o + o-hexa
1-pentadecanoyl-sn-glycero-3-phosphocholine
Pg 40:7
Andrastin a
Cholic acid
Pi 34:0
17.beta.-carboxy-17.alpha.-formyloxydexamethasone
Lithocholic acid
Pc(16:0e/10-hdohe)
Prostaglandin i2
15-cyclohexylpentanorprostaglandin f2.alpha.
Deoxycholic acid
L-alaninamide, n-[2-[2-(hydroxyamino)-2-oxoethyl]-4-methyl-1-oxopentyl]-3-(2-naphthalenyl)-l-alanyl-
Glycodeoxycholic acid
1-o-octyl-2-o-(n-methylcarbamoyl)-sn-glyceryl-3-phosphorylcholine
Taurochenodeoxycholate
Enterolactone
Pc(16:1e/17-hdohe)
7.alpha.,17.alpha.-dimethyl-5.beta.-androstane-3.alpha.,17.beta.-diol glucuronide
Codeine-6-.beta.-d-glucuronide
Stearoyl-coa
N-caffeoyl-o-methyltyramine
Novobiocin
8z,14z-eicosadienoic acid
7-dehydrocholesterol
Pseudouridine
18.beta.-glycyrrhetinic acid
His-ser
Specnuezhenide
Gentiopicroside
D-Maltose
Palatinose
Fructose 1-phosphate
Adenylosuccinate
Cytidine-5'-monophospho-n-acetylneuraminic acid
Eleutheroside e
Trans-3'-hydroxycotinine o-.beta.-d-glucuronide
3-(3-Hydroxyphenyl)propanoic acid
D-Fructose
Maltotriose
Hydroferulic acid
Linolenic acid
Pentasaccharides (hex-hex-hex-hex-hex)
Maltotetraose
D-Mannose
Phosphatidylcholine lyso alkyl 16:0
1,2-dioleoyl-sn-glycero-3-phosphoethanolamine-n,n-dimethyl
(2-aminoethoxy)[3-[hexadec-1-en-1-yloxy]-2-[icosa-5.8.11.14-tetraenoyloxy]propoxy]phosphinic acid
Pi(17:0/18-hepe)
N-fructosyl pyroglutamate
Cis,cis-muconic acid
N-glycolylneuraminic acid
Cis-4,7,10,13,16,19-docosahexaenoic acid
1-stearoyl-2-hydroxy-sn-glycero-3-phosphocholine
Arachidonic acid (peroxide free)
Pc(16:0e/8-hepe)
Linoleic acid
1,2-distearoyl-sn-glycero-3-phospho-l-serine
1-palmitoyl-2-oleoyl-phosphatidylglycerol
12(13)-epoxy-9z-octadecenoic acid
DSS1
DSS2
DSS3
DSS4
DSS5
GEN1
GEN2
GEN3
GEN4
GEN5
NC1
NC2
NC3
NC4
NC5
Group
DSS
GEN
NC
2
1
0
-1
-2

Figure S1. H, hierarchical clustering heat maps of metabolites with significant differences within groups under positive ion mode; I, hierarchical clustering heat map of metabolites with significant differences within groups under negative ion mode

S1J

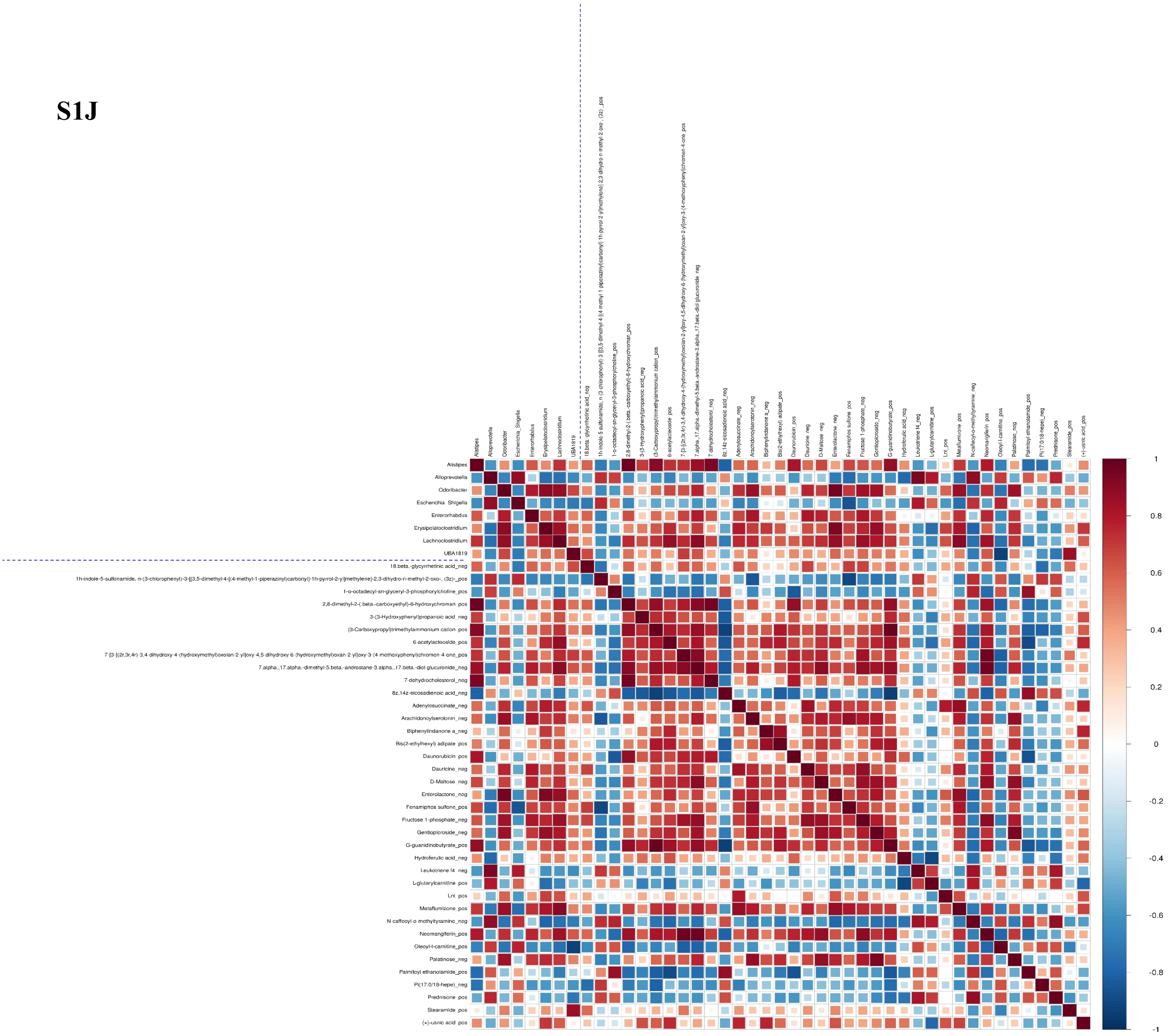

Alistipes
Alloprevotella
Odoribacter
Escherichia_Shigella
Enterorhabdus
Erysipelatoclostridium
Lachnoclostridium
UBA1819
18.beta.-glycyrrhetinic acid_neg
1h-indole-5-sulfonamide, n-(3-chlorophenyl)-3-[[3,5-dimethyl-4-[(4-methyl-1-piperazinyl)carbonyl]-1h-pyrrol-2-yl]methylene]-2,3-dihydro-n-methyl-2-oxo-, (3z)-_pos
1-o-octadecyl-sn-glyceryl-3-phosphorylcholine_pos
2,8-dimethyl-2-(.beta.-carboxyethyl)-6-hydroxychroman_pos
3-(3-Hydroxyphenyl)propanoic acid_neg
(3-Carboxypropyl)trimethylammonium cation_pos
6-acetylacteoside_pos
7-[3-[(2r,3r,4r)-3,4-dihydroxy-4-(hydroxymethyl)oxolan-2-yl]oxy-4,5-dihydroxy-6-(hydroxymethyl)oxan-2-yl]oxy-3-(4-methoxyphenyl)chromen-4-one_pos
7.alpha.,17.alpha.-dimethyl-5.beta.-androstane-3.alpha.,17.beta.-diol glucuronide_neg
7-dehydrocholesterol_neg
8z,14z-eicosadienoic acid_neg
Adenylosuccinate_neg
Arachidonoylserotonin_neg
Biphenylindanone a_neg
Bis(2-ethylhexyl) adipate_pos
Daunorubicin_pos
Dauricine_neg
D-Maltose_neg
Enterolactone_neg
Fenamiphos sulfone_pos
Fructose 1-phosphate_neg
Gentiopicroside_neg
G-guanidinobutyrate_pos
Hydroferulic acid_neg
Leukotriene f4_neg
L-glutarylcarnitine_pos
Lnt_pos
Metaflumizone_pos
N-caffeoyl-o-methyltyramine_neg
Neomangiferin_pos
Oleoyl-l-carnitine_pos
Palatinose_neg
Palmitoyl ethanolamide_pos
Pi(17:0/18-hepe)_neg
Prednisone_pos
Stearamide_pos
(+)-usnic acid_pos
1
0.8
0.6
0.4
0.2
0
-0.2
-0.4
-0.6
-0.8
-1

S1K

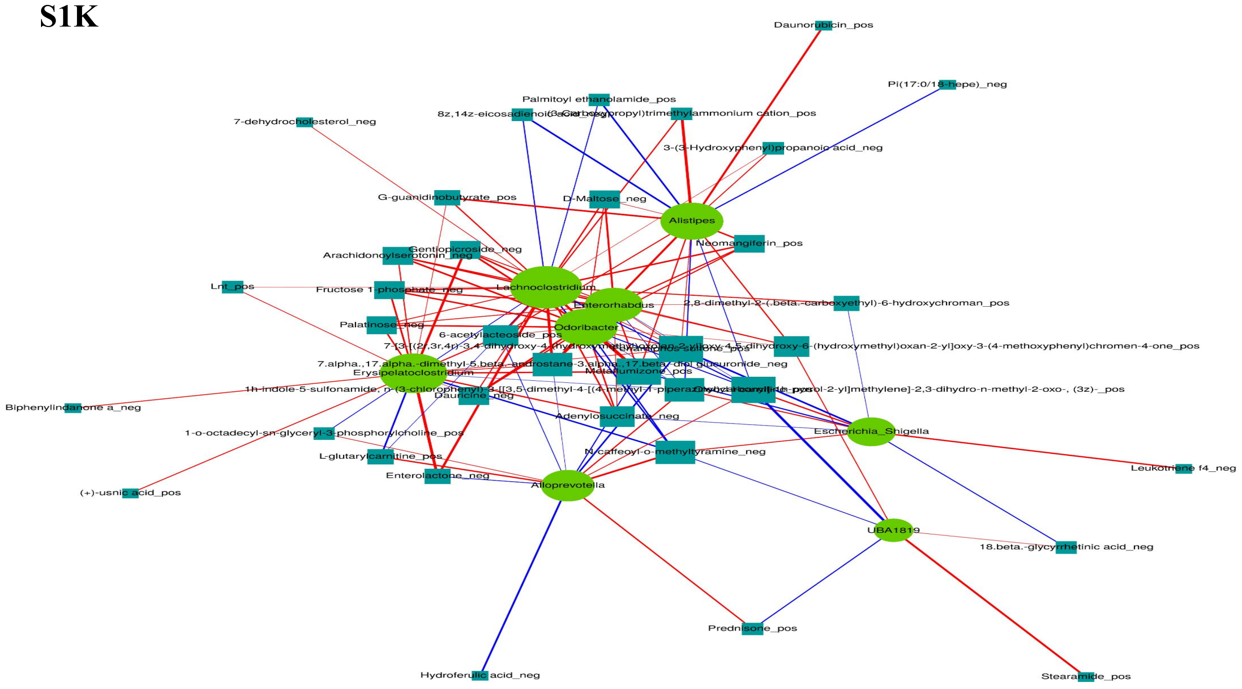

Daunorubicin_pos
Pi(17:0/18-hepe)_neg
Palmitoyl ethanolamide_pos
8z,14z-eicosadienoic acid_neg
(3-Carboxypropyl)trimethylammonium cation_pos
7-dehydrocholesterol_neg
3-(3-Hydroxyphenyl)propanoic acid_neg
G-guanidinobutyrate_pos
D-Maltose_neg
Alistipes
Neomangiferin_pos
Gentiopicroside_neg
Arachidonoylserotonin_neg
Lnt_pos
Fructose 1-phosphate_neg
Lachnoclostridium
Enterorhabdus
2,8-dimethyl-2-(.beta.-carboxyethyl)-6-hydroxychroman_pos
Palatinose_neg
Odoribacter
6-acetylacteoside_pos
7-[3-[(2r,3r,4r)-3,4-dihydroxy-4-(hydroxymethyl)oxolan-2-yl]oxy-4,5-dihydroxy-6-(hydroxymethyl)oxan-2-yl]oxy-3-(4-methoxyphenyl)chromen-4-one_pos
7.alpha.,17.alpha.-dimethyl-5.beta.-androstane-3.alpha.,17.beta.-diol glucuronide_neg
Erysipelatoclostridium
Metaflumizone_pos
1h-indole-5-sulfonamide, n-(3-chlorophenyl)-3-[[3,5-dimethyl-4-[(4-methyl-1-piperazinyl)carbonyl]-1h-pyrrol-2-yl]methylene]-2,3-dihydro-n-methyl-2-oxo-, (3z)-_pos
Dauricine_neg
Biphenylindanone a_neg
Adenylosuccinate_neg
1-o-octadecyl-sn-glyceryl-3-phosphorylcholine_pos
Escherichia_Shigella
L-glutarylcarnitine_pos
N-caffeoyl-o-methyltyramine_neg
Enterolactone_neg
Leukotriene f4_neg
(+)-usnic acid_pos
Alloprevotella
UBA1819
18.beta.-glycyrrhetinic acid_neg
Prednisone_pos
Hydroferulic acid_neg
Stearamide_pos

S1. J, heat map of the Spearman correlation coefficient matrix of significantly different microflora and significantly different metabolites; K, A network graph showing a Spearman analysis of the link between significantly distinct microbiota and significantly distinct metabolites
